# DNA damage-induced inhibition of rRNA synthesis by DNA-PK and PARP-1

**DOI:** 10.1093/nar/gkt502

**Published:** 2013-06-17

**Authors:** Anne S. Calkins, J. Dirk Iglehart, Jean-Bernard Lazaro

**Affiliations:** ^1^Department of Cancer Biology, Dana-Farber Cancer Institute, Boston, MA 02215, USA and ^2^Department of Surgery, Brigham and Women’s Hospital, Boston, MA 02115, USA

## Abstract

RNA synthesis and DNA replication cease after DNA damage. We studied RNA synthesis using an *in situ* run-on assay and found ribosomal RNA (rRNA) synthesis was inhibited 24 h after UV light, gamma radiation or DNA cross-linking by cisplatin in human cells. Cisplatin led to accumulation of cells in S phase. Inhibition of the DNA repair proteins DNA-dependent protein kinase (DNA-PK) or poly(ADP-ribose) polymerase 1 (PARP-1) prevented the DNA damage-induced block of rRNA synthesis. However, DNA-PK and PARP-1 inhibition did not prevent the cisplatin-induced arrest of cell cycle in S phase, nor did it induce *de novo* BrdU incorporation. Loss of DNA-PK function prevented activation of PARP-1 and its recruitment to chromatin in damaged cells, suggesting regulation of PARP-1 by DNA-PK within a pathway of DNA repair. From these results, we propose a sequential activation of DNA-PK and PARP-1 in cells arrested in S phase by DNA damage causes the interruption of rRNA synthesis after DNA damage.

## INTRODUCTION

Ribosomal RNA (rRNA) is synthesized in the nucleolus by RNA polymerase 1 (Pol1). Pol1 activity is dependent on the assembly of many separate proteins on rRNA gene promoters. The assembled complexes include the TATA binding protein (TBP), TBP-associated factors, Pol1, Transcription Factor II D (TFIID) and upstream binding factor (UBF) (1).

rRNA synthesis and DNA replication are inhibited following DNA damage by UV light, gamma radiation (IR) and genotoxic drugs such as cisplatin (2–4). Several proteins involved in DNA damage repair including poly(ADP-ribose) polymerase 1 (PARP-1), the DNA-dependent protein kinase (DNA-PK) subunit Ku (a heterodimer composed of Ku70 and Ku86), WRN and SSRP1 are present in the nucleolus and relocate to the nucleoplasm after damage (5–9). Ku binding to DNA ends recruits the catalytic subunit DNA-PKcs, forming the active DNA-PK holoenzyme (10). PARP-1 participates in base excision repair, homologous recombination and nonhomologous end-joining (NHEJ) and catalyzes the addition of poly(ADP-ribose) (PAR) to many targets including itself, PARP-2, histones, Ku and DNA-PKcs (11–13). DNA-PK initiates NHEJ of double strand breaks (DSBs) caused by genotoxic stress or V(D)J recombination. DNA-PK phosphorylates several substrates including its own subunits Ku and DNA-PKcs, histones and PARP-1 (14,15).

DNA-PK represses the Pol1 machinery of rRNA transcription *in vitro* (16,17). Pol1 activity present in cellular extracts increased in cells lacking DNA-PKcs or treated with wortmannin (18). Auto-phosphorylation of DNA-PK on its Ku subunit promotes displacement of the human SL1 transcription factor from the rDNA promoter region (18). DNA-PK is also able to phosphorylate TBP and TFIID (19). The role of PARP-1 in rRNA synthesis has been suggested. PARP was detected at the border of the dense fibrillar component of the nucleolus where rRNA transcription takes place (20). Nucleolar PARP-1 is lost in cells treated with transcription inhibitors (21,22). Laser micro-irradiation of the nucleus induced relocation of nucleolar PARP-1 into the nucleoplasm (6). In *Drosophila*, PARP-1 is necessary for nucleolar integrity and rRNA maturation (23). PARP-1 forms a complex with DNA-PK and the facilitator of chromatin transcription, FACT (24,25). These reports suggest a role for both DNA-PK and PARP-1 in rRNA synthesis following DNA damage.

## MATERIALS AND METHODS

### Cell culture and treatment

Human A2780 ovarian adenocarcinoma cells (Sigma) were cultured in Roswell Park Memorial Institute 1640 medium (RPMI) with 10% (v/v) fetal bovine serum, 100 U/ml penicillin and 100 µg/ml streptomycin (Invitrogen) at 37°C, 100% humidity and 5% CO_2_. A2780 cells expressing either nonsilencing small hairpin RNA (shRNA) or shRNA to DNA-PKcs were described elsewhere (5,25). To induce transient knockdown of PARP-1 in A2780 cells, lentiviral particles were produced in the Phx A packaging cell line by transfection with PCMV-dR8.91, pDM2-VSV-G (the RNAi consortium from the Broad Institute) and pLV vector coding for PARP-1 or GFP shRNA (Addgene). Exponentially growing A2780 cells were exposed to the filtered viral supernatant for 8 h. Medium was changed and incubated for 48 h, then cells were exposed to 2 µg/ml puromycin for 48 h before use in experiments.

Cells grown on dishes, 12-mm glass coverslips or multiwell glass slides (Electron Microscopy Sciences) using the DropArray system and Liquid Lid Sealing Fluid (Curiox Biosystems) were exposed to alpha-amanitin, actinomycin D, cisplatin (Sigma, dissolved in culture medium), wortmannin [all from Sigma, dissolved in dimethyl sulfoxide (DMSO)], Nu7026, Nu7441 (both from Selleck Chemicals, dissolved in DMSO), benzamide (Trevigen, dissolved in ethanol), olaparib (a kind gift from AstraZeneca, dissolved in DMSO) or the appropriate amount of solvent as indicated. Cells were exposed to UV-C light (254 nm) in a Stratagene UV Stratalinker 2400 or gamma radiation from an enclosed cesium source at an approximate rate of 1 Gray per minute.

### RNA and DNA synthesis

To monitor RNA synthesis, cells were incubated with 1 mM 5-ethynyl uridine (EU) (Invitrogen) in growth medium for 1 h, washed with phosphate-buffered saline at pH 7.4 (PBS), then fixed with 4% paraformaldehyde (PFA) for 10 min and permeabilized with methanol for 10 min at −20°C. The Click-iT reaction was carried out per manufacturer’s instructions (Invitrogen), followed by immunofluorescence for the nucleolar marker NOL1, detailed below. Total RNA was stained with SYTO RNASelect Green Fluorescent Cell Stain (Invitrogen) after PFA and methanol.

To monitor DNA synthesis, cells were exposed to 10 µM BrdU (Sigma) for 1 h in growth medium before fixation with PFA for 10 min and permeabilization with 4 N hydrochloric acid for 10 min. Immunofluorescence using mouse anti-BrdU (1:200, Sigma) was performed as described below.

### Immunofluorescence

To visualize nucleolar Ku, nucleolar PARP-1 or PAR, cells were permeabilized with a triton extraction solution [0.5% Triton X-100, 100 mM [piperazine-N,N′-bis(2-ethanesulfonic acid) (PIPES)] (pH 7.4), 1 mM EDTA, 2 mM MgCl_2_ and 300 mM sucrose] for 30 s before PFA fixation. To visualize phosphorylated DNA-PKcs, cells were fixed and permeabilized with methanol for 10 min at −20°C. Coverslips were incubated in PBS with 0.3% bovine serum albumin (BSA) and 0.1% sodium azide (PBS-BSA) for 20 min before incubation with primary antibodies.

The following primary antibodies were used: mouse anti-Ku70/Ku80 (1:400, Thermo Scientific), rabbit anti-PARP-1 (1:100, Cell Signaling), rabbit anti-NOL1 (1:400, Proteintech), rabbit anti-DNA-PKcs phosphorylated on serine 2056 (1:100, Abcam), mouse anti-PAR (1:200, clone 10H, Enzo Life Sciences) and mouse anti-B23 (1:400, Sigma). Antibodies were diluted in PBS-BSA and incubated with cells for 1 h at 37°C. Goat anti-rabbit and anti-mouse secondary antibodies conjugated to Alexa Fluor 488 or Alexa Fluor 594 fluorochromes (1:300, Invitrogen) were diluted in PBS-BSA and incubated with cells for 30 min at 37°C. Coverslips were mounted onto slides with VectaShield Mounting Medium with 4′,6-diamidino-2-phenylindole (DAPI) (Vector Laboratories).

### Cell lysate and chromatin preparation

Cells grown in dishes treated as indicated were harvested mechanically in PBS. Cell pellets were obtained by centrifugation and frozen at different time points. On thawing, pellets were incubated for 30 min at 4°C with lysis buffer [200 mM NaCl, 10% glycerol, 2 mM EDTA, 40 mM Tris (pH 8), 0.4% NP40] supplemented with proteasome inhibitor (Roche) and PhosSTOP phosphatase inhibitor cocktail (Roche). Lysates were cleared at 4°C for 10 min at 10 000 relative centrifugal force (RCF) and supernatants collected (soluble cell lysate). To obtain chromatin, remaining pellets were incubated twice for 5 min with lysis buffer at room temperature and washed once with micrococcal nuclease activity buffer [2 mM MgCl_2_, 1 mM CaCl_2_, 20 mM Tris (pH 8.0), 100 mM KCl]. Micrococcal nuclease (Roche) (0.05 U/µl) was added to resuspended pellets for 20 min at room temperature. Digested chromatin fractions were submitted to centrifugation at 20 000 RCF, and supernatants analyzed by gel electrophoresis and immunoblotting or stained with SimpleBlue Stain (Invitrogen).

### Immunoprecipitation

Immunoprecipitation was performed by incubating soluble cell lysates for 1 h at 4°C with 1 µg of mouse anti-DNA-PKcs antibody (NeoMarkers) followed by 1 h in the presence of 30 µl of G sepharose beads (GE Healthcare). Beads were washed with lysis buffer twice, and eluates obtained by incubation at 70°C for 10 min with NuPAGE sample loading buffer (Invitrogen).

### Western blotting and immunoblotting

Soluble cell lysates, chromatin fractions or immunoprecipitated protein samples were heated at 70°C for 10 min with NuPAGE sample loading buffer. Proteins separated by electrophoresis on a NuPAGE Bis-Tris 4–12% gradient gel (Novex) were transferred to a 0.45 µm pore nitrocellulose membrane (Biorad).

For immunoblotting, we used the following primary antibodies: mouse anti-Ku70 (1:2000, NeoMarkers), rabbit anti-PARP-1 (1:1000), rabbit anti-NOL1 (1:1000), rabbit anti-DNA-PKcs phosphorylated at serine 2056 (1:1000), mouse anti-β-actin (1:2000, Sigma), mouse anti-DNA-PKcs (1:1000), rabbit anti-ATM (1:2000, CalBiochem), mouse anti-Ku86 (1:1000, Cell Signaling) and rabbit anti-phosphorylated H2AX (1:1000, Cell Signaling). Goat anti-mouse and anti-rabbit secondary antibodies conjugated to HRP were used at 1:10 000 (Cell Signaling). All antibodies were diluted in PBS-0.05% Tween. Visualization was performed with a LAS-4000 Luminescent Image Analyzer after 3 min of incubation with the SuperSignal West Pico Reagent (Thermo Scientific).

### Image acquisition, quantification and analysis

For EU fluorescence, images were acquired at high magnification (630×) until 150–200 cells were imaged. All images were processed with CellProfiler [Broad Institute, Cambridge MA, (26), www.cellprofiler.org] using a program that identified nuclei and nucleoli from DAPI and anti-NOL1 or EU staining, respectively, and quantified fluorescence within each subcellular compartment. Nuclei were delineated by CellProfiler for use in figures (Supplementary Figure S1). For BrdU and anti-phosphorylated DNA-PKcs immunofluorescence, images were acquired at low magnification (100×), yielding >1000 imaged cells per experiment. All images were processed using a CellProfiler program that quantified BrdU or anti-phosphorylated DNA-PKcs fluorescence within DAPI-stained nuclei.

For anti-PAR immunofluorescence, fields were acquired at high magnification (630×) until 150–200 cells were imaged. To eliminate background fluorescence and sharpen foci, the Projections module in CellProfiler was used. Briefly, for each focused image, an additional two pictures were acquired above and below the most focused plane at an interval of 0.3 µm (z-stacking, AxioVision, Zeiss). All five images were loaded into the Projections module to reduce pixels with variable intensity. Using these processed images, the numbers of foci per nuclei were counted.

Three independent replicates were performed for each experiment acquired by fluorescent microscopy. Replicates were grouped for statistical analysis and comparisons made using one-way or two-way analysis of variance (ANOVA) tests in GraphPad Prism version 6 (www.graphpad.com). For all graphs, the average of the means from the three replicates with SEM is shown.

### Flow cytometry

Cells grown in dishes treated as indicated were washed twice with PBS and exposed to 0.08% trypsin (comp) for 5 min. Cells were suspended in PBS and centrifuged at 3000 RCF for 5 min. Cell pellets were suspended in PBS, then added dropwise to 70% ethanol at −20°C and fixed for at least 2 h on ice. Cells were centrifuged and suspended in PBS to eliminate ethanol. All samples were adjusted to 3 × 10^6^ cells/ml and suspended in PBS with 0.1% triton, 1 mg/ml RNase (Roche) and 0.1 mg/ml propidium iodide (Invitrogen) for 15 min at 37°C. Samples were stored at 4°C in the dark until analysis with a BD LSRFortessa cell analyzer (BD Biosciences). Collected data were interpreted using ModFit LT 3.3 software (Verity Software House).

## RESULTS AND DISCUSSION

### Analysis of rRNA synthesis after genotoxic stress

To investigate the regulation of rRNA synthesis by DNA-PK and PARP-1 after DNA damage, we performed run-on analysis of nascent rRNA synthesis. We analyzed EU incorporation into nascent RNA *in situ* by click chemistry (27). A2780 ovarian cancer cells incubated with EU for 60 min showed diffuse nucleoplasmic staining with prominent accumulation in the nucleoli. Treatment of cells with the Pol2 inhibitor alpha-amanitin blocked EU incorporation in the nucleoplasm. As expected, EU accumulation in the nucleoli was blocked by the Pol1 inhibitor actinomycin D (Supplementary Figure S2). Therefore, nucleolar EU incorporation was considered indicative of rRNA synthesis.

**Figure 1.**
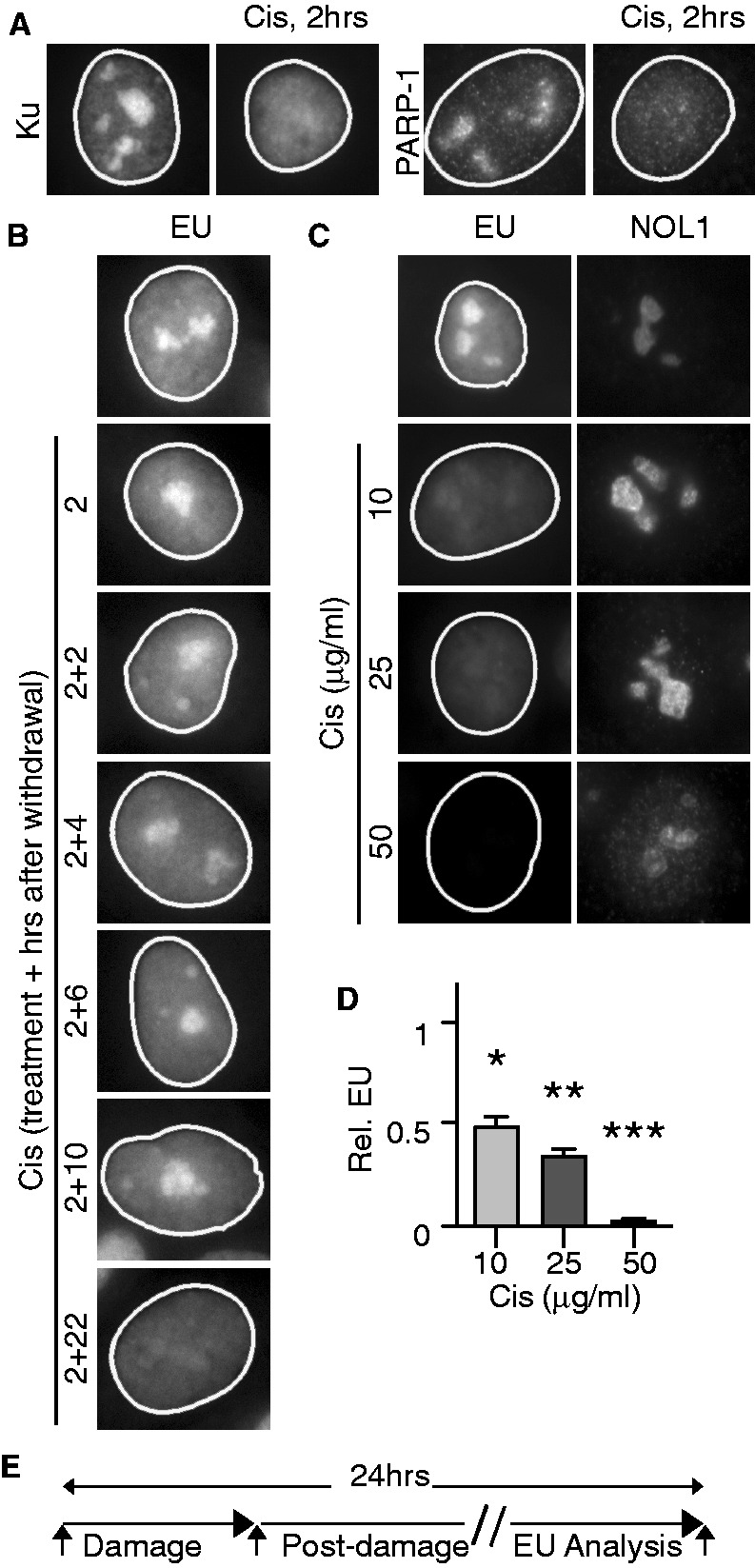
Inhibition of rRNA synthesis. (**A**) Nucleolar Ku and PARP-1 are lost after 2 h of treatment with 25 µg/ml cisplatin (Cis). Nuclei were outlined using CellProfiler software. (**B**) Cells were exposed to 25 µg/ml cisplatin for 2 h and analyzed for EU incorporation at indicated times after cisplatin withdrawal. (**C**) Inhibition of rRNA synthesis by cisplatin at 10, 25 and 50 µg/ml is shown by EU incorporation. Nucleoli were stained by anti-NOL1. (**D**) Quantification of adjusted nucleolar EU fluorescence shown in (C). Each condition was normalized to cells without cisplatin treatment. One-way ANOVA was followed by Dunnett’s test for comparison of each dose of cisplatin to the baseline. *, **, *** represent *P* ≤ 0.05, 0.01, 0.001, respectively. (**E**) Schematic of the sequence of steps in assays for RNA synthesis.

As previously reported, 2 h of treatment with cisplatin led to loss of nucleolar Ku (5) and similarly to loss of nucleolar PARP-1 (Figure 1A). For this reason, we analyzed the effect of a 2-h cisplatin pulse on rRNA synthesis. We measured incorporation of EU at various time points following this initial cisplatin treatment. Although no inhibition of RNA synthesis was found during the first 10 h following withdrawal of cisplatin, nucleolar RNA synthesis was blocked 22 h after the cisplatin pulse, concordant with the results of Jordan and Carmo-Fonseca (3) (Figure 1B). This effect was not due to alteration of the overall RNA content in the nucleolus, shown by total RNA staining (Supplementary Figure S3).

In similar experiments, we analyzed EU incorporation with the software CellProfiler to delineate nuclear, nucleolar and nucleoplasmic areas (Supplementary Figure S4A). Nucleolar RNA synthesis levels were inferred by subtracting average nucleoplasmic from average nucleolar EU signals (Supplementary Figure S4B). Significant inhibition of rRNA synthesis was observed from 10 to 25 µg/ml cisplatin, and all synthesis was abolished at 50 µg/ml (Figure 1C and D). Inhibition of rRNA synthesis was also observed 24 h after exposing cells to IR and UV light (Supplementary Figures S5A and S6A). Therefore, to study the potential role of PARP-1 and DNA-PK in the regulation of inhibition of rRNA synthesis after DNA damage, we performed analyses 22 h after 2 h treatment with cisplatin or 24 h after exposure to UV or IR (Figure 1E).

### DNA damage-induced inhibition of rRNA synthesis is dependent on the activities of both DNA-PK and PARP-1

**Figure 2.**
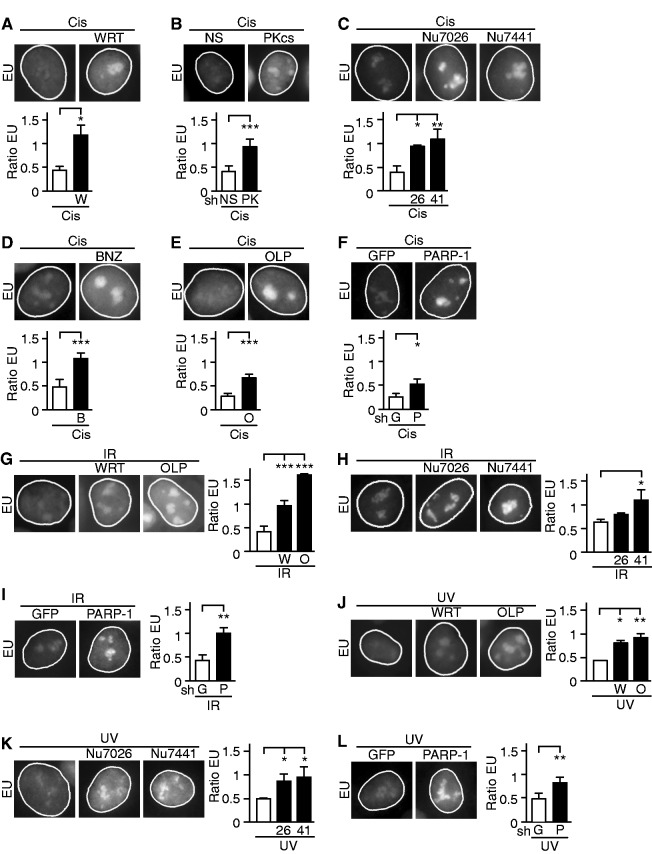
DNA damage-induced inhibition of rRNA synthesis is dependent on DNA-PK and PARP-1 activity. Representative nuclei stained by EU are shown 22 h after 2 h treatment with 25 µg/ml cisplatin. (**A–F**) Cells were treated with cisplatin under the following conditions: (A) pretreatment with wortmannin (WRT, W), (B) nonsilencing (NS) or DNA-PKcs (PKcs, PK) shRNA (sh), (C) pretreatment with Nu7026 (Nu7026, 26) or Nu7441 (Nu7441, 41), (D) pretreatment with benzamide (BNZ, B), (E) pretreatment with olaparib (OLP, O) or (F) GFP shRNA (G) or PARP-1 (P) shRNA. (**G–I**) Cells were treated with IR (2 Gray) under the following conditions: (G) pretreatment with wortmannin or olaparib, (H) pretreatment with Nu7026 or Nu7441 or (I) GFP or PARP-1 shRNA. (**J–L**) Cells were treated with UV-C (UV) at 5 J/m^2^ under the following conditions: (J) pretreatment with wortmannin or olaparib, (K) pretreatment with Nu7026 or Nu7441 or (L) GFP or PARP-1 shRNA. Before DNA damage, wortmannin was applied at 100 nM for 30 min, Nu7026 at 10 µM for 1 h, Nu7441 at 1 µM for 1 h, olaparib at 1 µM for 2 h and benzamide at 200 µM for 30 min. Accompanying quantifications of the adjusted nucleolar EU fluorescence are shown. Each condition was normalized to cells treated with DMSO or indicated inhibitors without DNA damaging treatment. Two-way ANOVA was followed by Holm-Sidak’s test for the comparison of cells with and without inhibitors. *, **, *** represent *P* ≤ 0.05, 0.01, 0.001, respectively.

We prevented the cisplatin-induced loss of nucleolar SSRP1 using the PI3K-like kinase family inhibitor wortmannin and shRNA against DNA-PKcs [(5), Supplementary Figure S7]. Treatment with wortmannin before addition of cisplatin significantly relieved inhibition of rRNA synthesis (Figure 2A and Supplementary Figure S8). In addition, silencing of DNA-PKcs expression by shRNA prevented cisplatin-induced inhibition of rRNA synthesis (Figure 2B and Supplementary Figure S9). Neither wortmannin nor expression of DNA-PKcs shRNA had visible effect on rRNA synthesis without cisplatin (Supplementary Figures S8A and S9A). As previously described, DNA-PKcs silencing resulted in lower levels of Ataxia Telangiectasia Mutated protein (ATM) [(28), Supplementary Figure S7]. Wortmannin may inhibit ATM or other PI3K-like kinase activities. For these reasons, we performed similar experiments using specific inhibitors of DNA-PK. Treatment with Nu7026 or Nu7441 before cisplatin significantly prevented inhibition of rRNA synthesis (Figure 2C and Supplementary Figure S10). Furthermore, specific inhibitors of the PI3K, ATM and Ataxia Telangiectasia and Rad3-Related protein (ATR) members of the PI3K family as well as C-Abl and p38 MAPK inhibitors did not significantly alter the inhibition of rRNA synthesis in cisplatin-treated cells (Supplementary Figure S11).

To inhibit PARP-1, we added either benzamide or olaparib to cells before cisplatin (29,30). Both inhibitors provided potent protection of rRNA synthesis (Figure 2D and E; Supplementary Figures S12 and S13). Neither benzamide nor olaparib interfered with rRNA synthesis when added to the cells without cisplatin (Supplementary Figures S12A and S13A). Similarly, transient silencing of PARP-1 by shRNA (Supplementary Figure S14) prevented cisplatin-induced inhibition of rRNA synthesis (Figure 2F and Supplementary Figure S15). Western blot analysis showed that prevention of the block of rRNA synthesis by chemical inhibition of DNA-PK and PARP-1 was not due to changes in the overall protein levels of Ku70, Ku86, DNA-PKcs and PARP-1 (Supplementary Figure S16). Notably, as with DNA-PK, PARP-1 silencing led to lower levels of ATM (Supplementary Figure S14).

We next investigated the roles of PARP-1 and DNA-PK in the UV- and IR-dependent block of rRNA synthesis. Cells were irradiated with increasing doses of gamma radiation (2–10 Gray) or UV-C light (5–20 J/m^2^). Following either mode of damage, rRNA synthesis was significantly reduced at all tested doses. Pretreatment with wortmannin, olaparib, Nu7026, Nu7441 and PARP-1 shRNA protected rRNA synthesis after both UV light and gamma radiation (Figure 2G–L; Supplementary Figures S5, S6 and S17–S20). The block of rRNA synthesis was not relieved at higher doses of gamma or UV radiation (10 Gray and 20 J/m^2^), similar to cisplatin at 50 µg/ml. As with cisplatin, cellular machinery may be nonspecifically disrupted at high doses of genotoxic agents.

The inhibition of nascent nucleoplasmic RNA synthesis was also observed after DNA damage, as expected. However, results were inconclusive regarding the role of DNA-PK and PARP-1 in synthesis of new RNA in the nucleoplasm (data not shown). Our results suggest DNA-PK and PARP-1 are not necessary for rRNA synthesis under normal circumstances. After genotoxic stress, these proteins participate in mechanisms inhibiting rRNA synthesis that are common to several and possibly all types of DNA damage.

In human cells, DNA-PK but not ATM inhibited mRNA synthesis 1 h after a single DSB, and mRNA synthesis was restored within 6 h (31). In mouse embryo fibroblasts (MEFs), rRNA synthesis was inhibited 20 min after IR and restored at 60 min. Inhibition of rRNA synthesis was prevented in ATM−/− but not Ku−/− MEFs (32). Although the discrepancy between the requirement for DNA-PK in the first study and ATM in the latter may arise from the study of mRNA and rRNA, respectively, it is notable that the studies were performed in human and rodent cells, respectively. Primate cells exhibit ∼50 times more DNA-PK activity than rodent cells due to higher expression of DNA-PKcs (15). Furthermore, rRNA synthesis is unaltered in PARP-1−/− MEFs (22). A reconciliation of these studies, along with results presented herein, suggests specific roles for PARP-1 and DNA-PK in higher mammals.

Many targets of DNA-PK and PARP-1 could mediate repression of rRNA synthesis. PARP-1 or DNA-PK interacts with or affects Pol1, FACT, B23/nucleophosmin, polycomb repressive complex, UBF, TBP, TIP5 and transcription initiation factor SL1, all of which have been implicated in rRNA synthesis (5,16–18,24,33–40). Circumstantially, it seems unlikely that DNA-PK and PARP-1 inhibit rRNA synthesis through only one target. Although DNA-PK and PARP-1 can control the Pol1 machinery directly or indirectly, they may be targeting each other.

### DNA-PK acts upstream of PARP-1 to block rRNA synthesis after DNA damage

**Figure 3.**
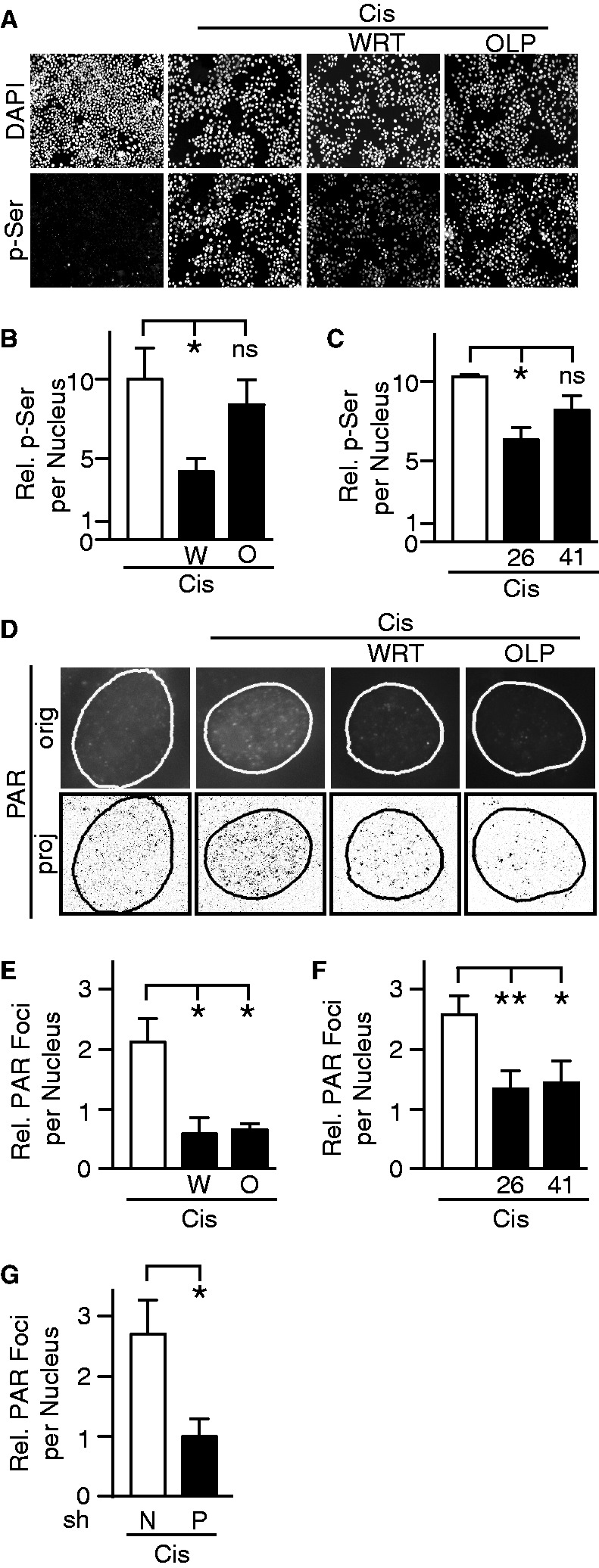
Cisplatin-induced PARP-1 activity is DNA-PK dependent. (**A**) Immunofluorescence after treatment with cisplatin and wortmannin or olaparib as described in Figure 2. Representative fields stained for DAPI and DNA-PKcs p-Ser^2056^ (p-Ser). (**B**) Quantification of the average p-Ser fluorescence per nucleus from experiments using wortmannin and olaparib or (**C**) Nu7026 and Nu7441. (**D**) Immunofluorescence of PAR after treatment with cisplatin and wortmannin or olaparib as described in Figure 2. Supplementary Figure S13 describes image production; the original image (orig) and the processed image (proj) are shown. (**E**) Quantification of the average relative number of PAR foci per nucleus is shown from experiments using wortmannin and olaparib, (**F**) Nu7026 and Nu7441 or (**G**) in cells expressing either nonsilencing or DNA-PKcs shRNA. Each condition was normalized to cells treated with DMSO but not cisplatin. One-way ANOVA was followed by Holm–Sidak’s test to compare responses to cisplatin of cells exposed to wortmannin or olaparib or expressing DNA-PKcs shRNA versus cells exposed to DMSO or expressing NS shRNA. *, ** represent *P* ≤ 0.05, 0.01, respectively; ns indicates nonsignificant result (*P* > 0.05).

**Figure 4.**
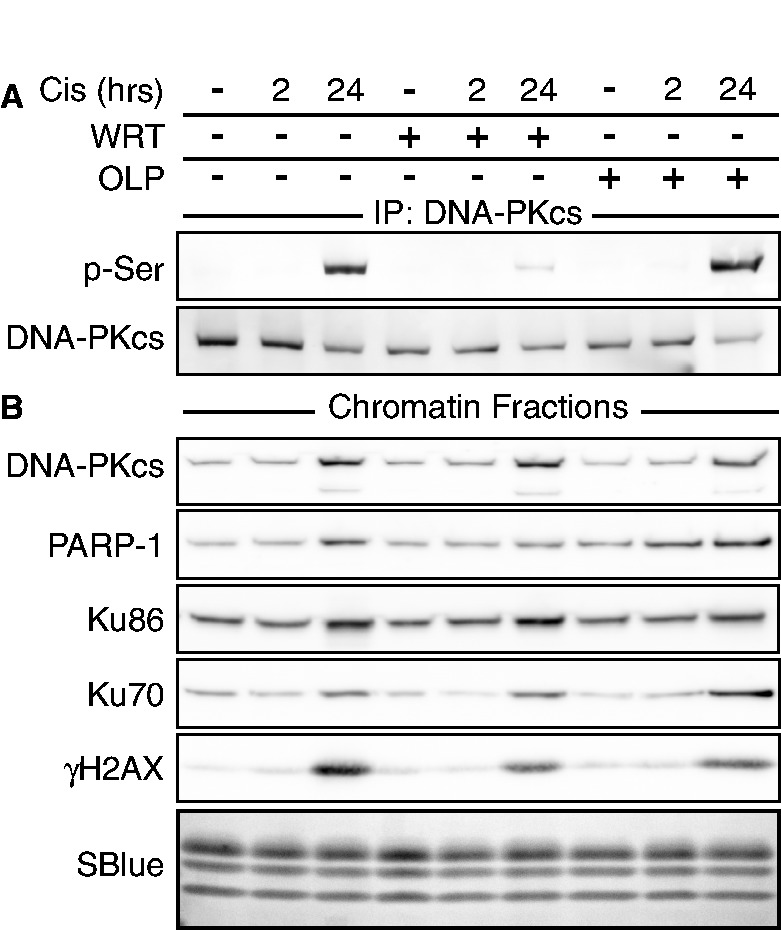
Cisplatin-induced recruitment of PARP-1 and DNA-PK to chromatin. Drug treatments are indicated at the top of both panels. (**A**) Immunoprecipitation of DNA-PKcs and immunoblot of DNA-PKcs p-Ser^2056^. (**B**) Immunoblot of chromatin fractions. SimpleBlue staining of histones is shown as loading control (SBlue).

We measured levels of auto-phosphorylation of DNA-PKcs at serine 2056 (DNA-PKcs p-Ser^2056^) by immunofluorescence before and after cisplatin treatment. Twenty-two hours after cisplatin treatment, we observed a significant signal increase of DNA-PKcs p-Ser^2056^ inside nuclei (Figure 3A). DNA-PK activation was significantly inhibited in cells pretreated with wortmannin and Nu7026 (Figure 3B–C). Confirmation of inhibition of DNA-PK by Nu7441 required western blotting analysis (Supplementary Figure S21). Olaparib pretreatment had no effect on DNA-PK activation (Figure 3A and B), which was verified by western blotting (Figure 4A). These results suggest PARP-1 does not affect DNA-PK activation after DNA damage. Interestingly, PARP-1 inhibition leads to activation of DNA-PK in BRCA2-deficient cells but not in cells with wild-type BRCA2 (41).

PARP-1 activation leads to accumulation of PAR on substrate proteins. We performed immunofluorescence using an anti-PAR antibody and analyzed the images to identify and enumerate PAR nuclear foci (Supplementary Figure S22). Twenty-two hours after cisplatin, treated cells exhibited increased number of PAR foci when compared with untreated cells. No increase in the number of PAR foci was observed with olaparib pretreatment, verifying the identity of the PAR staining. Kinase inhibition by wortmannin as well as specific inhibition of DNA-PK by Nu7026 and Nu7441 resulted in lack of PAR accumulation after cisplatin (Figure 3D–F). Similarly, cells expressing shRNA for DNA-PKcs exhibited reduced cisplatin-induced accumulation of PAR when compared with cells expressing nonsilencing shRNA (Figure 3G). DNA-PK–dependent activation of PARP-1 was also seen 30 min after addition of high doses of cisplatin (Supplementary Figure S23A–E). When studying nucleolar trafficking, DNA-PKcs depletion prevents loss of nucleolar PARP-1 (Supplementary Figure S23F–G). Thus, DNA-PK might be regulating PARP-1 directly or through a cascade of events. Our results establish PARP-1 activation is DNA-PK dependent and strongly suggest DNA-PK acts upstream of PARP-1 to inhibit rRNA synthesis after DNA damage.

### DNA-PK and PARP-1 recruitment to chromatin

We performed immunoprecipitation of DNA-PKcs in soluble cell lysates and analyzed DNA-PKcs p-Ser^2056^. We found DNA-PK activation after cisplatin was prevented by wortmannin but not olaparib, corroborating immunofluorescence results shown earlier (Figure 4A).

Events on chromatin after DNA damage were examined. Chromatin-associated H2AX was not phosphorylated at 2 h, but strikingly phosphorylated 22 h after the cisplatin pulse. This effect was mitigated in samples incubated with either wortmannin or olaparib before cisplatin (Figure 4B). During the same timeframe, DNA-PK and PARP-1 were recruited to chromatin. DNA-PKcs, Ku86 and Ku70 were recruited to chromatin regardless of the activation of DNA-PK. However, recruitment of PARP-1 to chromatin was diminished by wortmannin pretreatment. The recruitment of these proteins was not substantially affected if cells were preincubated with olaparib (Figure 4B). Recruitment of DNA-PK to chromatin suggests the appearance of breaks in DNA. Recruitment of PARP-1, or its continued presence on chromatin, was regulated by DNA-PK, providing further evidence of PARP-1 regulation by DNA-PK.

### Inhibition of PARP-1 and DNA-PK do not alter DNA damage-induced block of DNA replication

**Figure 5.**
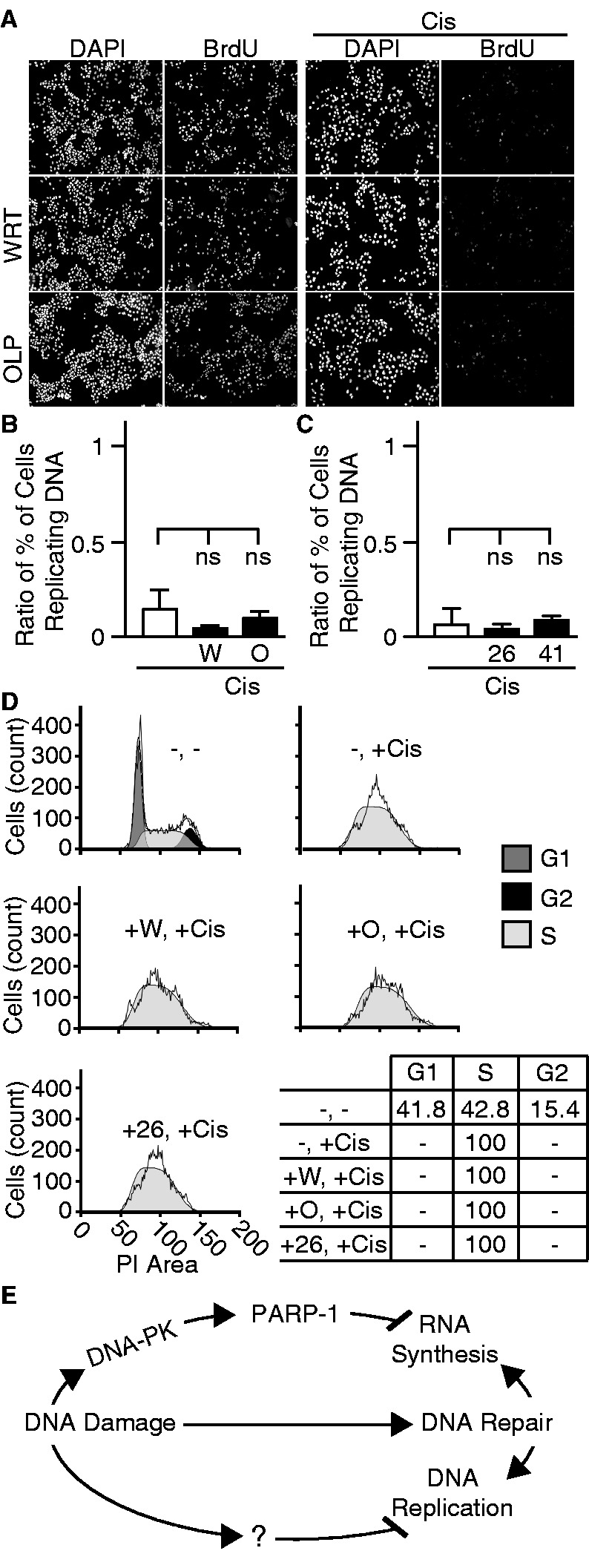
Regulation of rRNA synthesis is distinct from DNA synthesis. (**A**) Immunofluorescence of BrdU incorporation was performed in cells treated with cisplatin, wortmannin and olaparib as in Figure 2. Representative fields stained for DAPI and BrdU. (**B**) Quantification of BrdU incorporation expressed as ratios of total cells actively replicating DNA from experiments using wortmannin and olaparib or (**C**) Nu7026 and Nu7441. Each condition was normalized to cells treated with DMSO or indicated inhibitors without cisplatin treatment. Decrease in nuclear BrdU significantly differs from the baseline after two-way ANOVA test followed by Dunnett’s test (*P* ≤ 0.001), while cisplatin-treated values were determined by separate two-way ANOVA followed by Holm–Sidak’s test (*P* > 0.05, ns). (**D**) Histograms plotting the area of propidium iodide staining (PI) and number of cells as determined by flow cytometry. G1, G2 and S phases were shaded by ModFit. Percentage of cells in G1, G2 and S phases is included as a table. (**E**) Schematic depicting the involvement of DNA-PK and PARP-1 in DNA damage-induced inhibition of rRNA synthesis.

Inhibition of rRNA synthesis 24 h after DNA damage could be due to a cell cycle effect. Both the cell cycle and DNA replication are inhibited by genotoxic stress (42). We monitored BrdU incorporation *in situ* by adding BrdU 1 h before fixation to determine the fraction of cells with ongoing DNA replication. Twenty-two hours after the cisplatin pulse, DNA replication was significantly reduced. To determine if inhibition of PARP-1 or DNA-PK induced resumption of the cell cycle, cells were treated with cisplatin and inhibitors as in Figure 2. In contrast to rRNA synthesis, DNA replication was blocked even with inhibition of PARP-1 and DNA-PK (Figure 5A–C). To determine in which phase of the cell cycle cisplatin treatment induced arrest, we performed flow cytometry. As previously described by Sorenson and Eastman, we found cells treated with cisplatin arrested in S phase (43). Pretreatment with inhibitors did not change the phase at which arrest occurred (Figure 5D and Supplementary Figure S24). Cisplatin-induced block of rRNA synthesis is prevented by DNA-PK and PARP-1 inhibition without altering cell cycle withdrawal in S phase. Thus, the control of rRNA synthesis by DNA-PK and PARP-1 after DNA damage is uncoupled from regulation of the cell cycle (Figure 5E).

Nucleolar exit of Ku and PARP-1 occurs in the first 2 h after DNA damage. We observed inhibition of rRNA synthesis between 12 and 24 h after cisplatin, UV light and IR. These results suggest the relationship between nucleolar exit and inhibition of rRNA synthesis is indirect. A common product of these damaging insults could be DNA DSBs, which induce DNA-PK activation. Indeed, in all experimental conditions, the cells accumulated broadly throughout S phase (Figure 5D). DSBs appear when replication forks stall after genotoxic stress in cells whose exit from S phase is blocked (44). This suggests the broad bell shape of the S phase distribution in damaged cells reflects various times at which replication forks stalled at points of damage. At the time of replication, PARP-1 inhibits rRNA synthesis by maintaining inherited silencing of rDNA genes by chromatin modification (34). Furthermore, inhibition of Pol1 by DNA-PK only occurs in the presence of DNA ends, simulating DSBs (17). The production of DSBs results in recruitment of DNA-PK and PARP-1 to the chromatin and to their activation (15,45). It is possible these events are necessary for inhibition of rRNA synthesis after DNA damage. In conclusion, we observe DNA-PK acts upstream of PARP-1 to block rRNA synthesis after DNA damage. A possible mechanism involves stalled forks accumulating in S phase that result in DSBs, which initiate this pathway.

## SUPPLEMENTARY DATA

Supplementary Data are available at NAR Online: Supplementary Figures 1–24 and Supplementary References [46–51].

## FUNDING

Pallotta Investigator Fund (to J.B.L.); the Susan F. Smith Center for Women’s Cancers; the Marjorie Powell Allen Memorial Fund and Barbara and Paul Ferry. Funding for open access charge: The Pallotta Investigator Fund.

*Conflict of interest statement.* None declared.

## Supplementary Material

Supplementary Data
