# Supplementary material for: DNA damage-induced inhibition of rRNA synthesis by DNA-PK and PARP-1
Source: Nucleic Acids Res. 2013 Jun 17;41(15):7378–86. doi: 10.1093/nar/gkt502 (PMC3753630; doi:10.1093/nar/gkt502)
Supplement: Supplementary Data [file supp_41_15_7378__index.html]

DNA damage-induced inhibition of rRNA synthesis by DNA-PK and PARP-1 — DNA damage-induced inhibition of rRNA synthesis by DNA-PK and PARP-1 — Supplementary Data 

# DNA damage-induced inhibition of rRNA synthesis by DNA-PK and PARP-1

## Supplementary Data

files

**Files in this Data Supplement:**

- Supplementary Data - pdf file
