# Supplementary material for: DNA damage-induced inhibition of rRNA synthesis by DNA-PK and PARP-1
Source: Nucleic Acids Res. 2013 Jun 17;41(15):7378–86. doi: 10.1093/nar/gkt502 (PMC3753630; doi:10.1093/nar/gkt502)
Supplement: Supplementary Data [file supp_gkt502_nar-03349-d-2012-File007.pdf]

Supplemental Figure 1

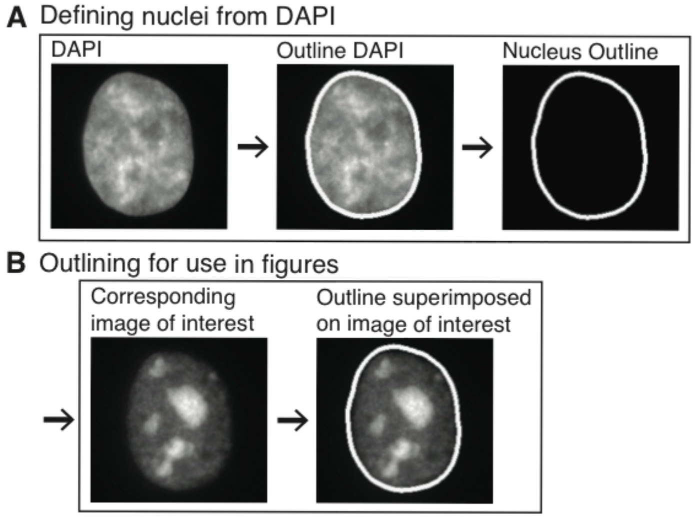

CellProfiler provides outlines of nuclei for figures. CellProfiler identifies nuclei from DAPI staining based on threshold brightness levels above background, diameter restrictions, and shape. (**A**) CellProfiler outlines an identified nucleus and retains the outline. (**B**) The nuclear outline is superimposed on Ku staining in the same cell.

Supplemental Figure 2

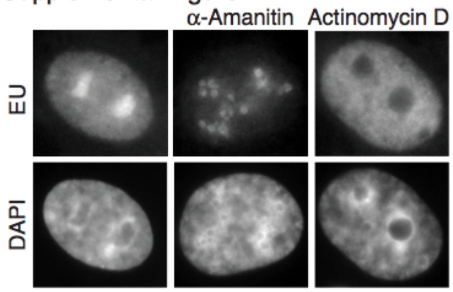

EU incorporation after exposure to α-amanitin (25 μg/ml for 4 hours) or actinomycin D (5 nM for 4 hours). DAPI-stained nuclei are shown below each representative nucleus.

Supplemental Figure 3

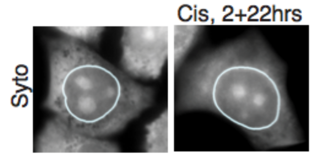

Staining for total RNA by Syto RNASelect in untreated cells or 22 hours after 2 hours treatment with 25 μg/ml cisplatin. Nuclei are outlined.

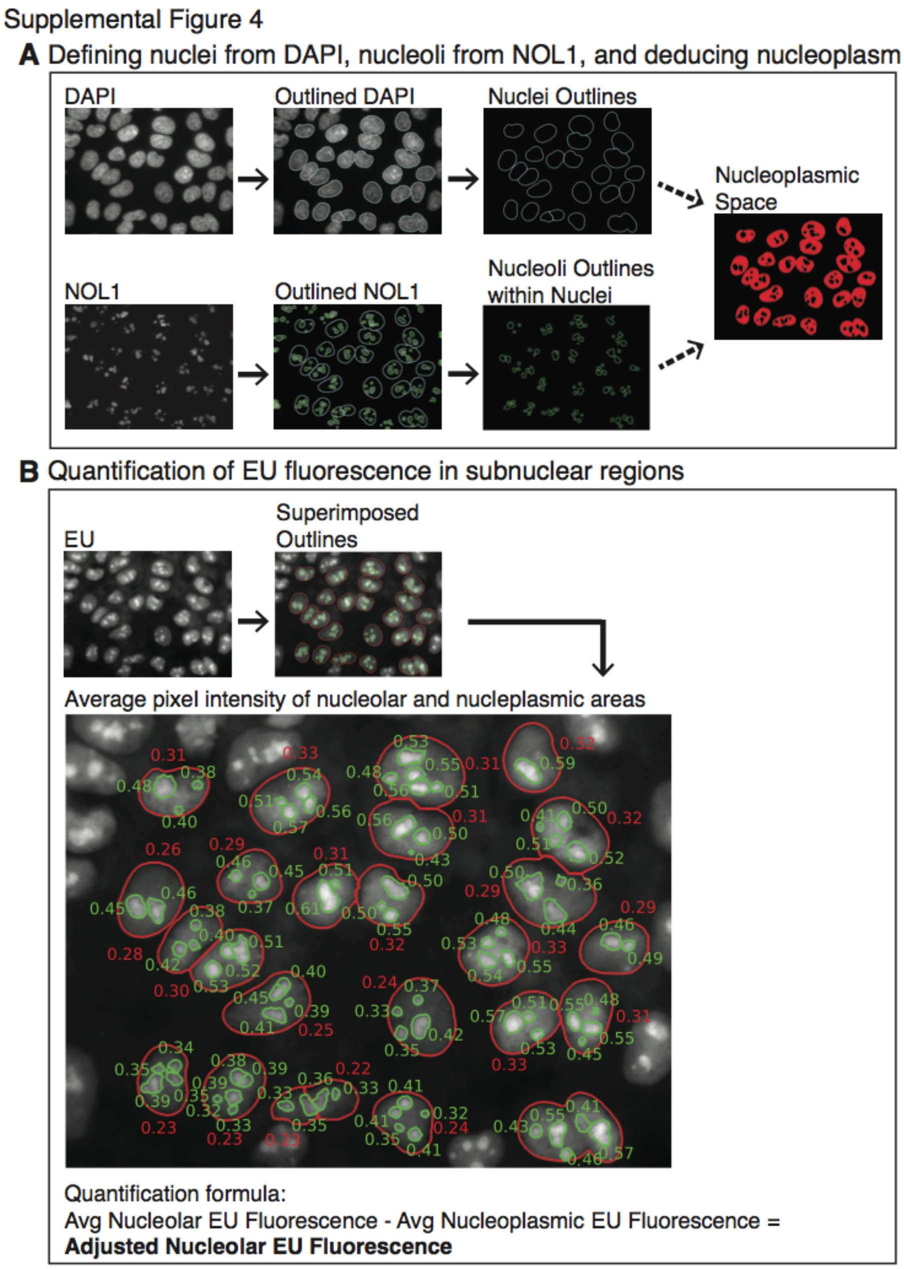

Supplemental Figure 4. CellProfiler quantifies fluorescence through shape-based recognition. (**A**) CellProfiler identifies nuclei from DAPI staining as described in Supplemental Figure S1. Nuclei that touch the edge of the field are excluded. DAPI-stained nuclei are outlined (Outlined DAPI) and nuclear space is defined (Nuclei Outlines). Similarly, CellProfiler identifies nucleoli from anti-NOL1 staining based on threshold, size, and shape (Outlined NOL1). Only nucleoli within previously identified nuclei are retained (Nucleoli Outlines within Nuclei). Finally, the area occupied by nucleoli is subtracted from the nuclear area to define the nucleoplasm (Nucleoplasmic Space, shown in red). (**B**) Quantification of EU fluorescence in subnuclear regions. Outlines of the nucleoplasmic area (red) and outlines of nucleoli (green) are superimposed on the corresponding EU stains. The average pixel intensity per nucleolus (green numbers) and nucleoplasmic area (red numbers) is calculated and shown for demonstration. By subtracting the average nucleoplasmic intensity from average nucleolar intensity, the adjusted nucleolar EU fluorescence is obtained.

Supplemental Figure 5

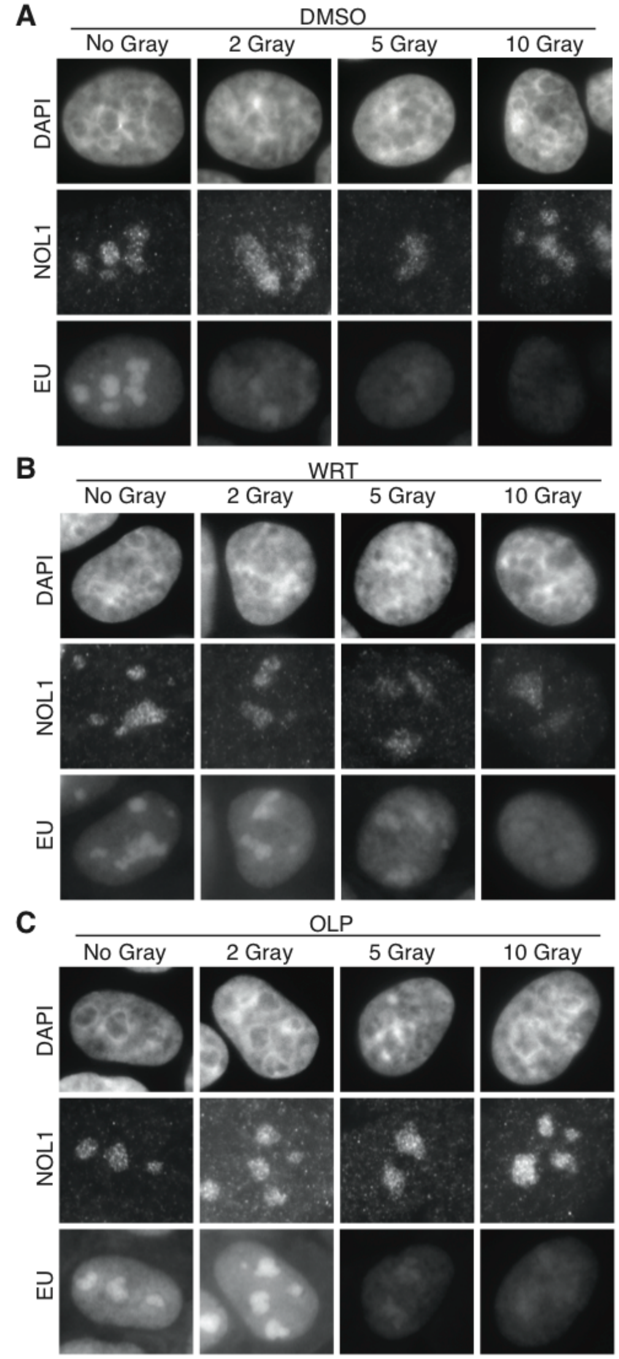

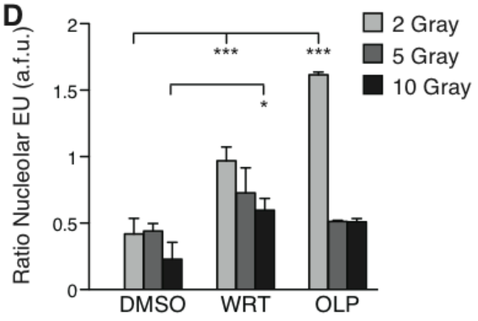

Dose-response of gamma radiation-induced inhibition of rRNA synthesis following pretreatment with wortmannin or olaparib. (**A**) IR doses of 2, 5, or 10 Gray inhibit rRNA synthesis shown by EU incorporation. Nuclei and nucleoli were stained by DAPI and anti-NOL1, respectively. (**B**) Cells exposed to wortmannin (WRT). (**C**) Cells exposed to olaparib (OLP). In both panels, inhibitor exposure was done as in Figure 2. (**D**) Quantification of adjusted nucleolar EU fluorescence. Each condition was normalized to cells treated with DMSO or indicated inhibitors without IR treatment. Two-way ANOVA was performed followed by Holm-Sidak’s test to compare the response of cells exposed to inhibitors versus cells exposed to DMSO. *, *** represent $p \leq 0.05$, 0.001, respectively. Only statistically significant results are shown.

Supplemental Figure 6

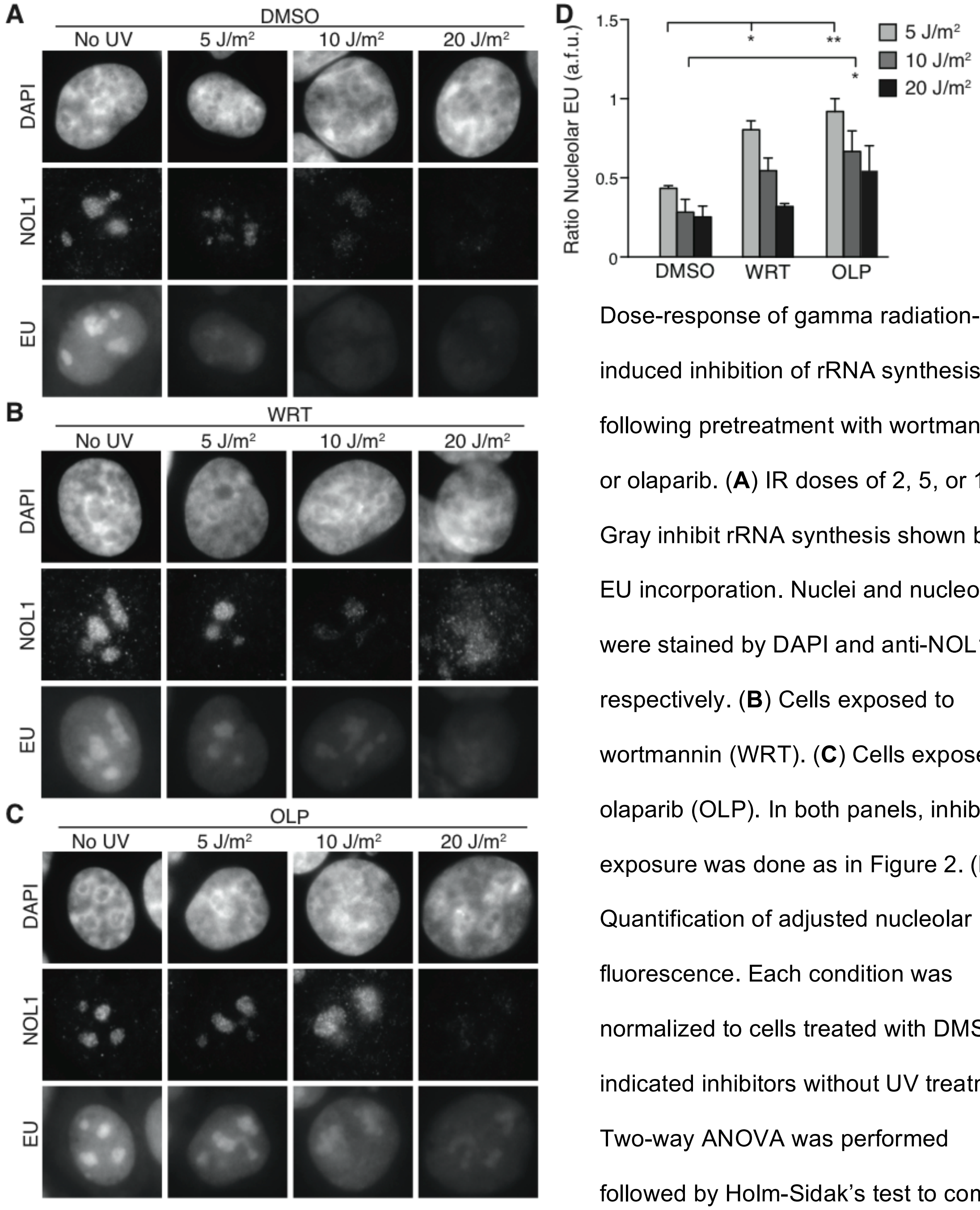

Dose-response of gamma radiation-induced inhibition of rRNA synthesis following pretreatment with wortmannin or olaparib. (**A**) IR doses of 2, 5, or 10 Gray inhibit rRNA synthesis shown by EU incorporation. Nuclei and nucleoli were stained by DAPI and anti-NOL1, respectively. (**B**) Cells exposed to wortmannin (WRT). (**C**) Cells exposed to olaparib (OLP). In both panels, inhibitor exposure was done as in Figure 2. (**D**) Quantification of adjusted nucleolar EU fluorescence. Each condition was normalized to cells treated with DMSO or indicated inhibitors without UV treatment. Two-way ANOVA was performed followed by Holm-Sidak's test to compare the response of cells exposed to inhibitors versus cells exposed to DMSO. *, ** represent $p \leq 0.05$, 0.001, respectively. Only statistically significant results are shown.

Supplemental Figure 7

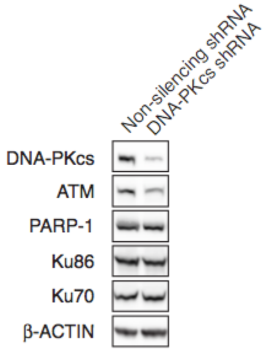

Immunoblotting analysis of soluble proteins in cells stably expressing non-silencing shRNA or shRNA to DNA-PKcs.

Supplemental Figure 8

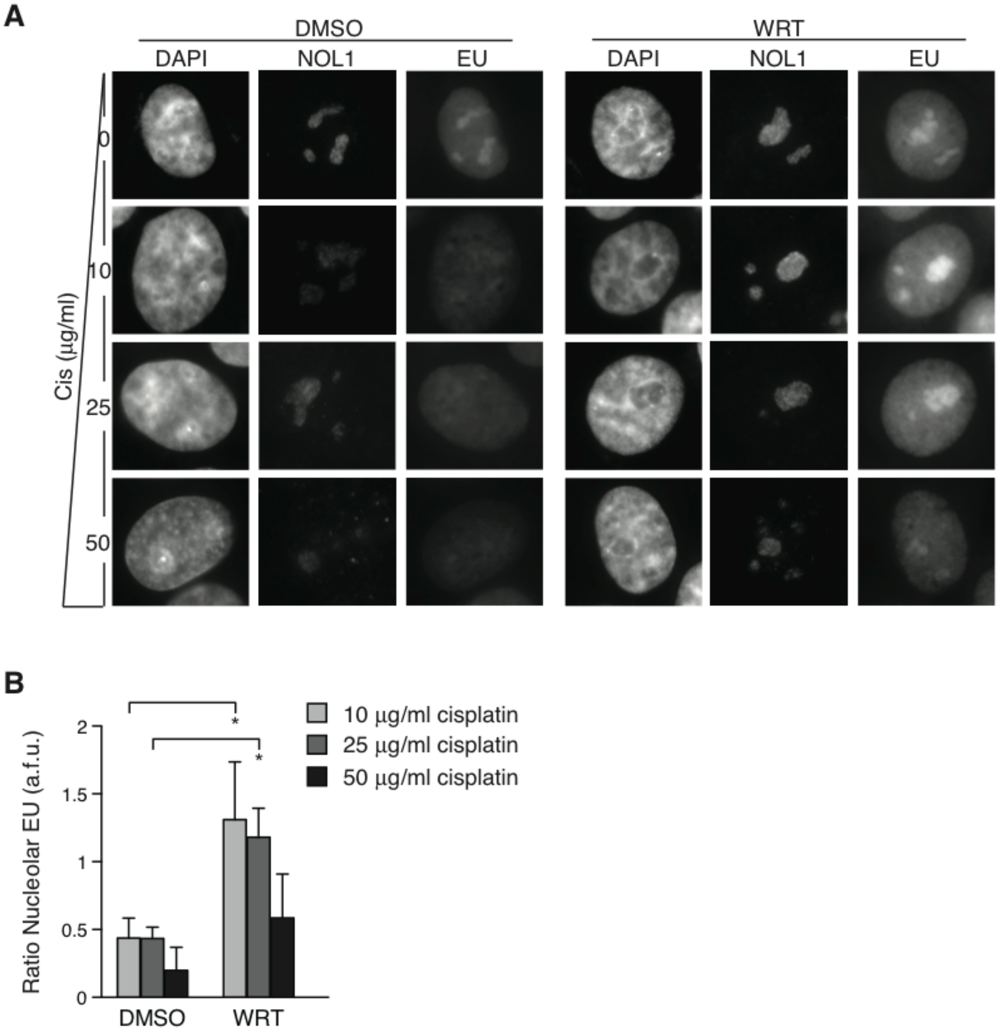

Dose-response of cisplatin-induced inhibition of rRNA synthesis following pretreatment with wortmannin. (**A**) Cisplatin at 10, 25, or 50 µg/ml inhibits rRNA synthesis shown by EU incorporation. Nuclei and nucleoli were stained by DAPI and anti-NOL1, respectively. Cells exposed to wortmannin (WRT) prior to 10 and 25 µg/ml cisplatin do not exhibit marked inhibition of rRNA synthesis. (**B**) Quantification of adjusted nucleolar EU fluorescence. Each condition was normalized to cells treated with DMSO or wortmannin without cisplatin treatment. Two-way ANOVA was performed followed by Holm-Sidak's test to compare the response of cells exposed to wortmannin versus cells exposed to DMSO at each concentration of cisplatin. * represents $p \leq 0.05$. Only statistically significant results are shown.

Supplemental Figure 9

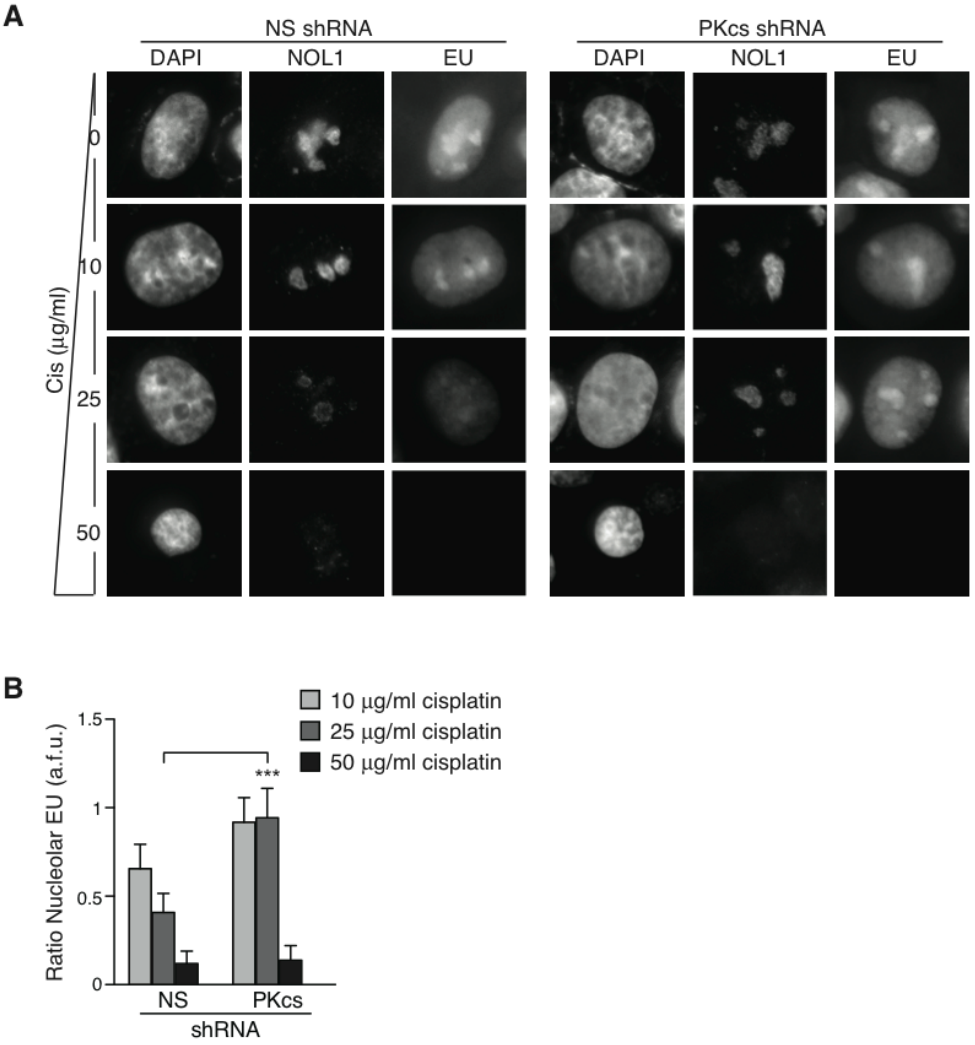

Dose-response of cisplatin-induced inhibition of rRNA synthesis in cells expressing non-silencing (NS) or PKcs shRNA. (**A**) Cisplatin at 10, 25, or 50 µg/ml inhibits rRNA synthesis shown by EU incorporation. Nuclei and nucleoli were stained by DAPI and anti-NOL1, respectively. Cells expressing shRNA to PKcs do not exhibit marked inhibition of rRNA synthesis at cisplatin doses of 10 and 25 µg/ml. (**B**) Quantification of adjusted nucleolar EU fluorescence. Each condition was normalized to cells expressing the corresponding shRNA without cisplatin treatment. Two-way ANOVA was performed followed by Holm-Sidak's test to compare the response of cells expressing PKcs shRNA versus cells expressing NS shRNA at each concentration of cisplatin. *** represents $p \leq 0.001$. Only statistically significant results are shown.

Supplemental Figure 10

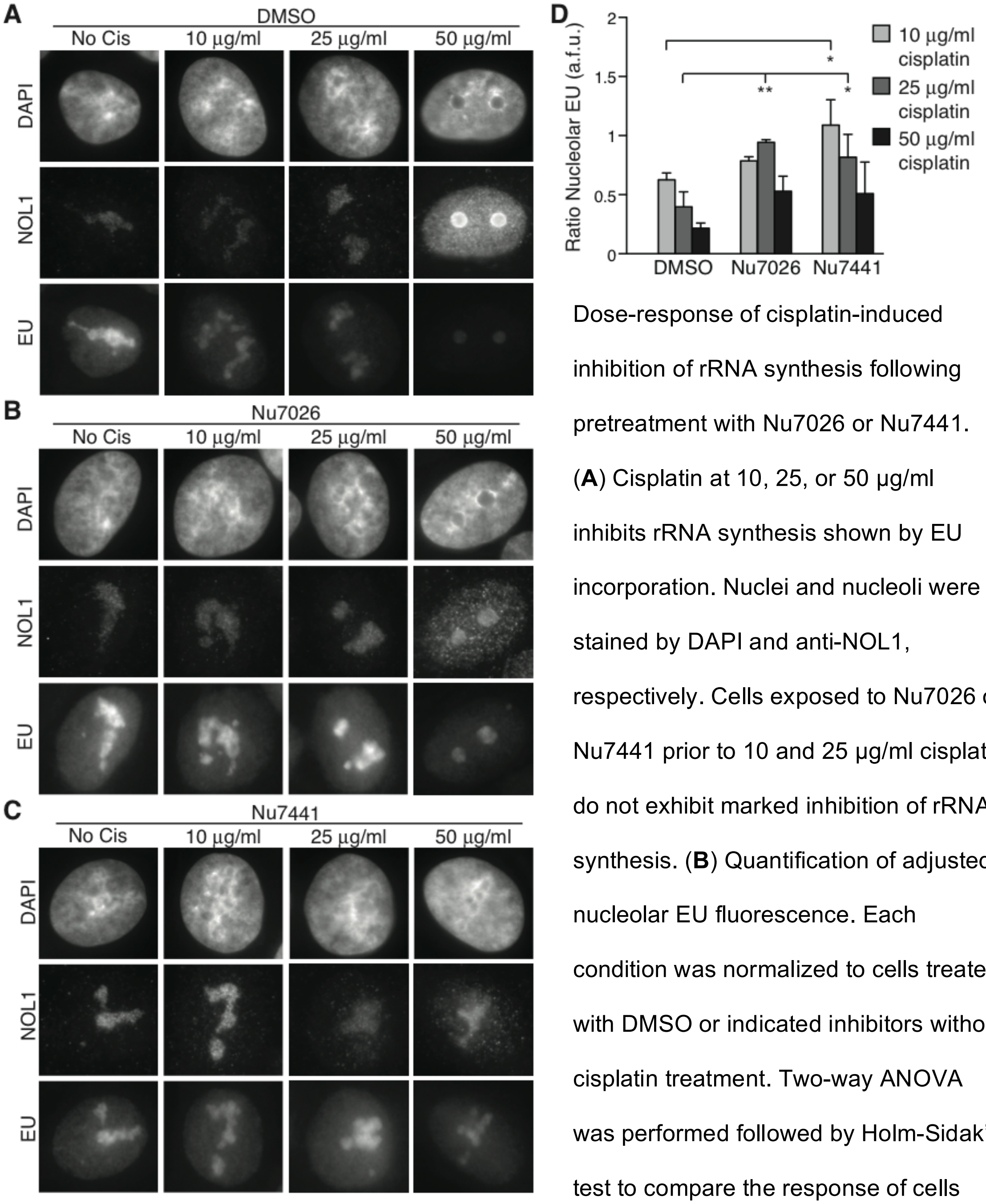

Dose-response of cisplatin-induced inhibition of rRNA synthesis following pretreatment with Nu7026 or Nu7441. (**A**) Cisplatin at 10, 25, or 50 μg/ml inhibits rRNA synthesis shown by EU incorporation. Nuclei and nucleoli were stained by DAPI and anti-NOL1, respectively. Cells exposed to Nu7026 or Nu7441 prior to 10 and 25 μg/ml cisplatin do not exhibit marked inhibition of rRNA synthesis. (**B**) Quantification of adjusted nucleolar EU fluorescence. Each condition was normalized to cells treated with DMSO or indicated inhibitors without cisplatin treatment. Two-way ANOVA was performed followed by Holm-Sidak's test to compare the response of cells exposed to inhibitors versus cells exposed to DMSO at each concentration of cisplatin. *, ** represents $p \leq 0.05, 0.01$, respectively. Only statistically significant results are shown.

Supplemental Figure 11

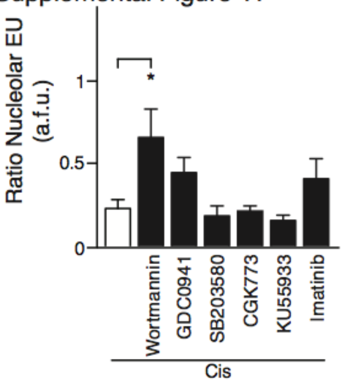

Drug	Concentration	Target	p value	Reference
Wortmannin	100 nM	PI3K-like kinases	0.0233	Sarkaria et al, 46
GDC0941	75 nM	PI3K	0.4724	Folkes et al, 47
SB203580	500 nM	p38 MAPK	0.9239	Lali et al, 48
CGK773	200 nM	ATM/ATR	0.9239	Goldstein et al, 49
KU55933	13 nM	ATM	0.9186	Hickson et al, 50
Imatinib	2 μM	c-Abl	0.6063	Buchdunger et al, 51

46. Folkes, A.J., Ahmadi, K., Alderton, W.K., Alix, S., Baker, S.J., Box, G., Chuckowree, I.S., Clarke, P.A., Depledge, P., Eccles, S.A. *et al.* (2008) The identification of 2-(1H-indazol-4-yl)-6-(4-methanesulfonyl-piperazin-1-ylmethyl)-4-morpholin-4-yl-t hieno[3,2-d]pyrimidine (GDC-0941) as a potent, selective, orally bioavailable inhibitor of class I PI3 kinase for the treatment of cancer. *J Med Chem*, **51**, 5522-5532.
47. Sarkaria, J.N., Tibbetts, R.S., Busby, E.C., Kennedy, A.P., Hill, D.E. and Abraham, R.T. (1998) Inhibition of phosphoinositide 3-kinase related kinases by the radiosensitizing agent wortmannin. *Cancer Res*, **58**, 4375-4382.
48. Lali, F.V., Hunt, A.E., Turner, S.J. and Foxwell, B.M. (2000) The pyridinyl imidazole inhibitor SB203580 blocks phosphoinositide-dependent protein kinase activity, protein kinase B phosphorylation, and retinoblastoma hyperphosphorylation in interleukin-2-stimulated T cells independently of p38 mitogen-activated protein kinase. *J Biol Chem*, **275**, 7395-7402.
49. Goldstein, M., Roos, W.P. and Kaina, B. (2008) Apoptotic death induced by the cyclophosphamide analogue mafosfamide in human lymphoblastoid cells: contribution of DNA replication, transcription inhibition and Chk/p53 signaling. *Toxicol Appl Pharmacol*, **229**, 20-32.
50. Hickson, I., Zhao, Y., Richardson, C.J., Green, S.J., Martin, N.M., Orr, A.I., Reaper, P.M., Jackson, S.P., Curtin, N.J. and Smith, G.C. (2004) Identification and characterization of a novel and specific inhibitor of the ataxia-telangiectasia mutated kinase ATM. *Cancer Res*, **64**, 9152-9159.
51. Buchdunger, E., Zimmermann, J., Mett, H., Meyer, T., Muller, M., Druker, B.J. and Lydon, N.B. (1996) Inhibition of the Abl protein-tyrosine kinase in vitro and in vivo by a 2-phenylaminopyrimidine derivative. *Cancer Res*, **56**, 100-104.

Screen of kinase inhibitors for effects on rRNA synthesis. Quantification of adjusted nucleolar EU fluorescence in experiments performed as in Figure 2. Each condition was normalized to cells treated with DMSO or indicated inhibitors without cisplatin treatment. Two-way ANOVA was performed followed by Holm-Sidak's test to compare the response of cells exposed to DMSO versus cells exposed to inhibitors. * represents $p \leq 0.05$. Only statistically significant results are shown on the graph, but all p-values are included in the table. Cells were exposed to all inhibitors for 1 hour except wortmannin, for which exposure was 30 minutes. The targets and concentrations used for each drug are shown. Concentrations were chosen from the literature indicated (46-51).

Supplemental Figure 12

A

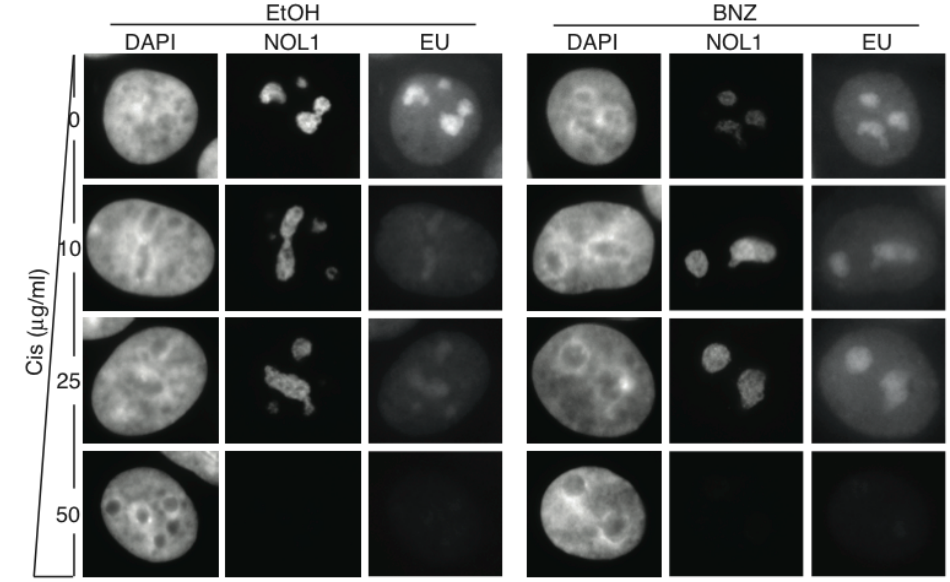

B

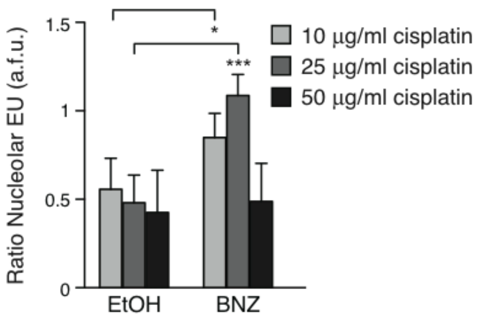

Dose-response of cisplatin-induced inhibition of rRNA synthesis following pretreatment with benzamide. (**A**) Cisplatin at 10, 25, or 50 µg/ml inhibits rRNA synthesis shown by EU incorporation. Nuclei and nucleoli were stained by DAPI and anti-NOL1, respectively. Cells exposed to benzamide (BNZ) prior to 10 and 25 µg/ml cisplatin do not exhibit marked inhibition of rRNA synthesis. (**B**) Quantification of adjusted nucleolar EU fluorescence. Each condition was normalized to cells treated with ethanol or benzamide without cisplatin treatment. Two-way ANOVA was performed followed by Holm-Sidak's test to compare the response of cells exposed to benzamide versus cells exposed to ethanol. *, *** represent $p \leq 0.05$, 0.001, respectively. Only statistically significant results are shown.

Supplemental Figure 13

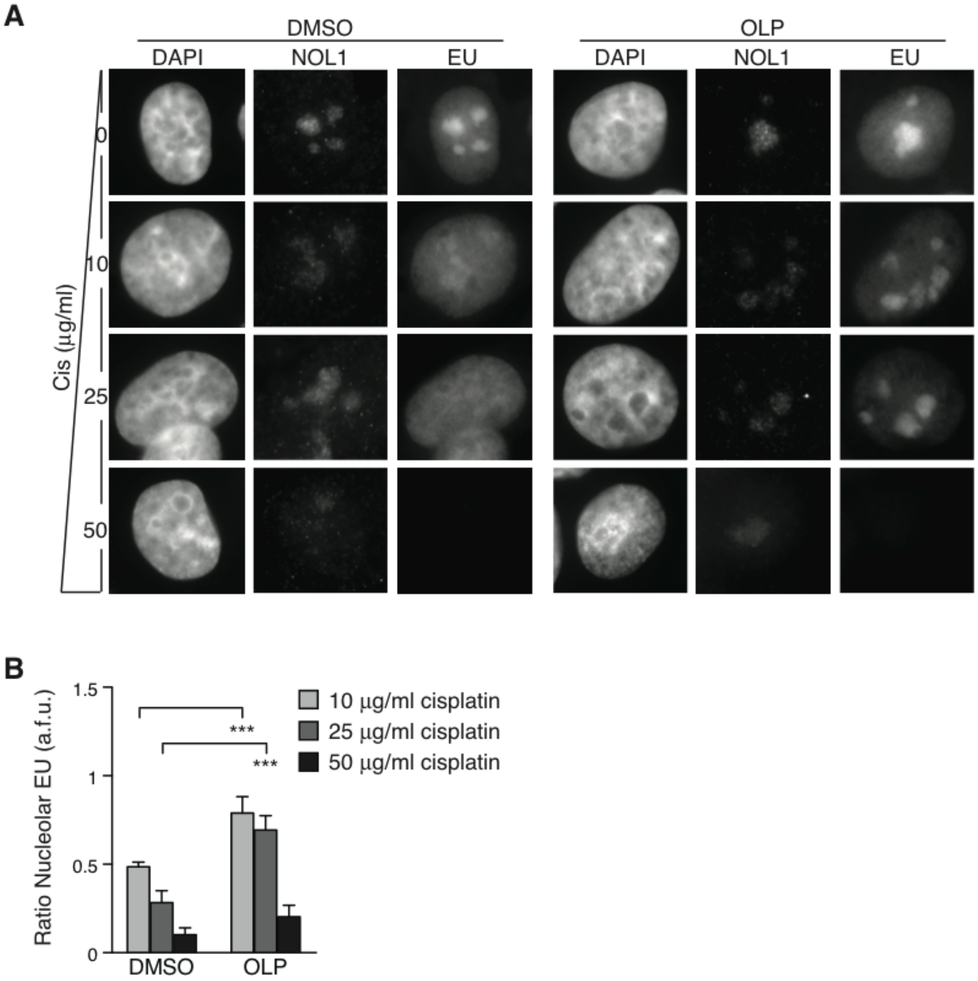

Dose-response of cisplatin-induced inhibition of rRNA synthesis following pre-treatment with olaparib. (**A**) Cisplatin at 10, 25, or 50 µg/ml inhibits rRNA synthesis shown by EU incorporation. Cells exposed to olaparib (OLP) prior to 10 and 25 µg/ml cisplatin do not exhibit marked inhibition of rRNA synthesis. Nuclei and nucleoli were stained by DAPI and anti-NOL1, respectively. (**B**) Quantification of adjusted nucleolar EU fluorescence. Each condition was normalized to cells treated with DMSO or olaparib without cisplatin treatment. Two-way ANOVA was performed followed by Holm-Sidak's test to compare the response of cells exposed to DMSO versus cells exposed to olaparib. *** represents $p \leq 0.001$. Only statistically significant results are shown.

Supplemental Figure 14

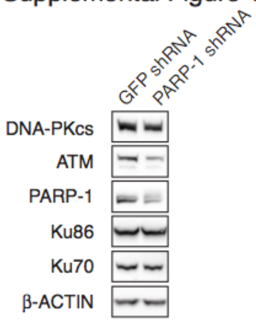

Immunoblotting analysis of soluble proteins in cells transiently expressing shRNA to GFP or PARP-1.

Supplemental Figure 15

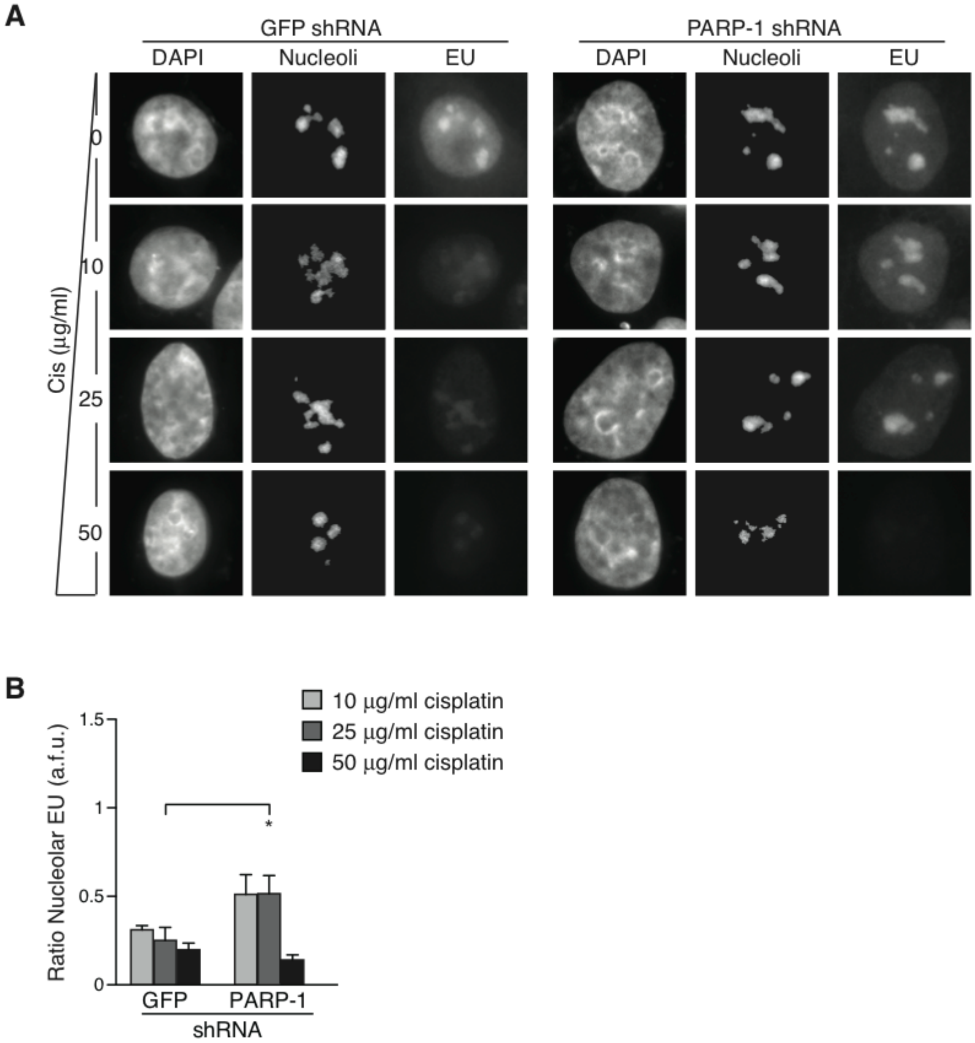

Dose-response of cisplatin-induced inhibition of rRNA synthesis in cells expressing GFP or PARP-1 shRNA. (**A**) Cisplatin at 10, 25, or 50 µg/ml inhibits rRNA synthesis shown by EU incorporation. Nuclei were stained by DAPI, and CellProfiler was used to define Nucleoli from EU staining. (**B**) Quantification of adjusted nucleolar EU fluorescence. Each condition was normalized to cells expressing the corresponding shRNA without cisplatin treatment. Two-way ANOVA was performed followed by Holm-Sidak's test to compare the response of cells expressing PARP-1 shRNA versus cells expressing GFP shRNA at each concentration of cisplatin. * represents $p \leq 0.05$. Only statistically significant results are shown.

Supplemental Figure 16

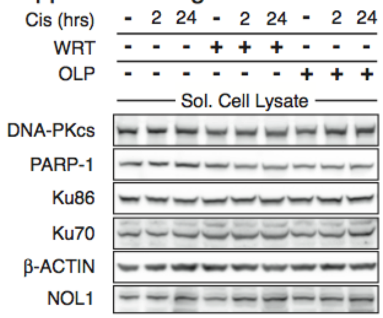

Immunoblotting analysis of soluble proteins after exposure to cisplatin (Cis), wortmannin (WRT) and olaparib (OLP) as in Figure 4.

Supplemental Figure 17

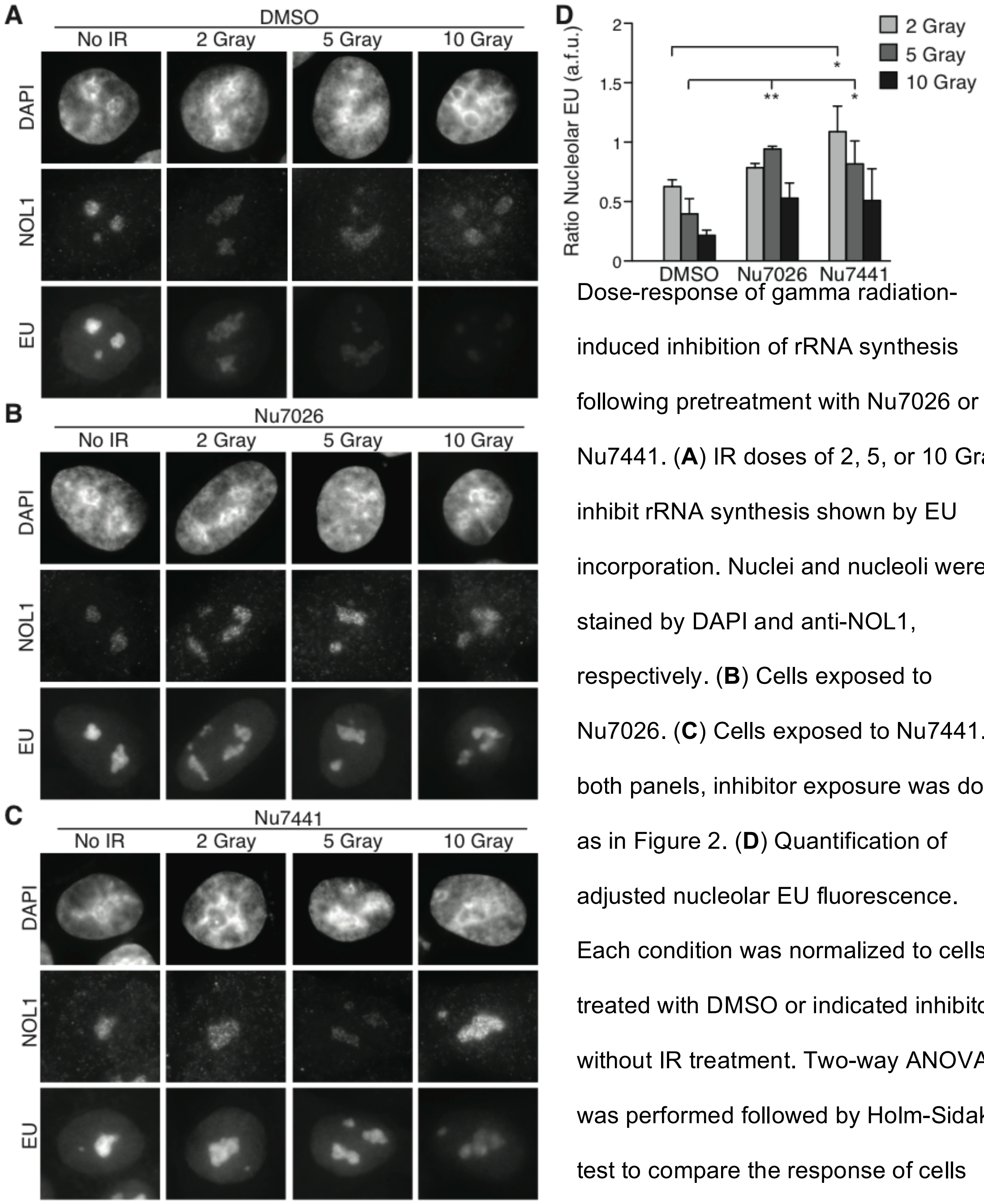

Dose-response of gamma radiation-induced inhibition of rRNA synthesis following pretreatment with Nu7026 or Nu7441. (**A**) IR doses of 2, 5, or 10 Gray inhibit rRNA synthesis shown by EU incorporation. Nuclei and nucleoli were stained by DAPI and anti-NOL1, respectively. (**B**) Cells exposed to Nu7026. (**C**) Cells exposed to Nu7441. In both panels, inhibitor exposure was done as in Figure 2. (**D**) Quantification of adjusted nucleolar EU fluorescence. Each condition was normalized to cells treated with DMSO or indicated inhibitors without IR treatment. Two-way ANOVA was performed followed by Holm-Sidak's test to compare the response of cells exposed to inhibitors versus cells exposed to DMSO. *, ** represent p ≤ 0.05, 0.01, respectively. Only statistically significant results are shown.

Supplemental Figure 18

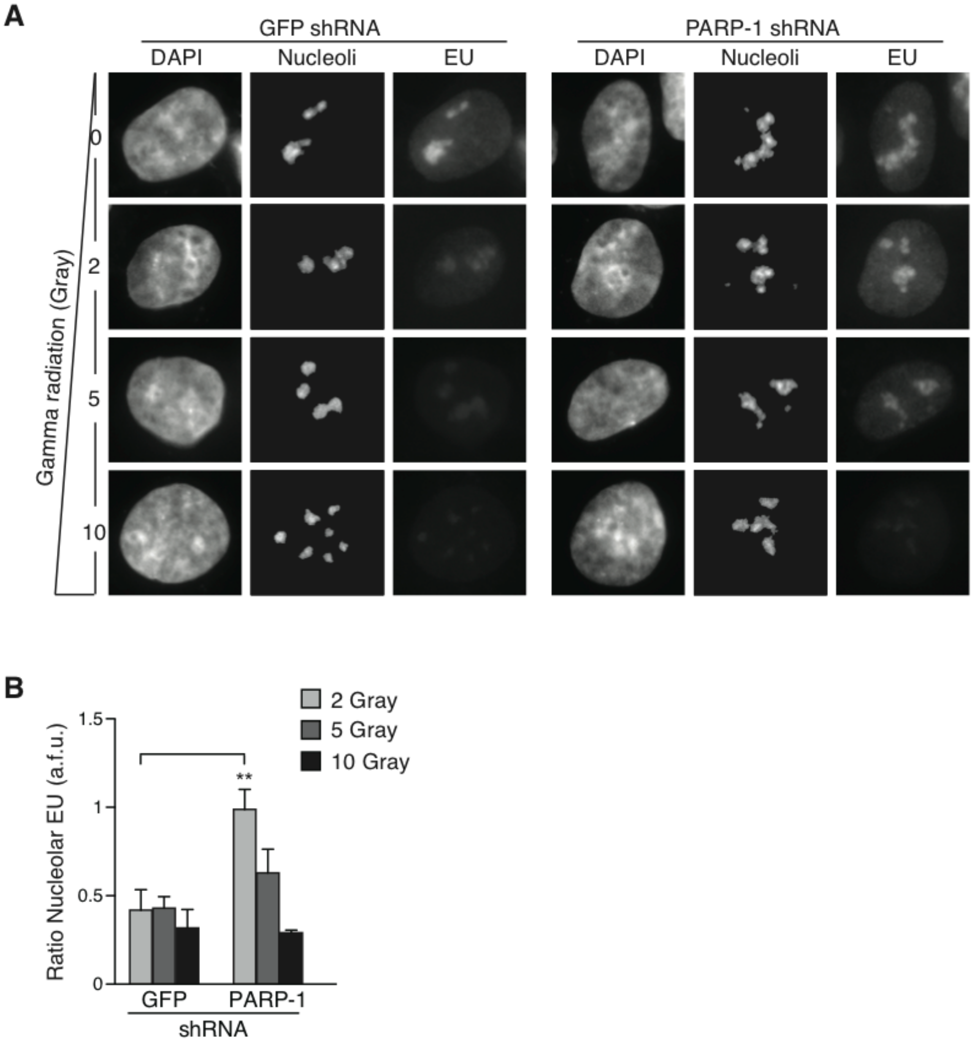

Dose-response of gamma radiation-induced inhibition of rRNA synthesis in cells expressing GFP or PARP-1 shRNA. (**A**) IR doses of 2, 5, or 10 Gray inhibit rRNA synthesis shown by EU incorporation. Nuclei were stained by DAPI, and CellProfiler was used to define Nucleoli from EU staining. Cells expressing shRNA to PARP-1 do not exhibit marked inhibition of rRNA synthesis at IR dose of 2 Gray. (**B**) Quantification of adjusted nucleolar EU fluorescence. Each condition was normalized to cells expressing the corresponding shRNA without IR treatment. Two-way ANOVA was performed followed by Holm-Sidak's test to compare the response of cells expressing PARP-1 shRNA versus cells expressing GFP shRNA at each dose of IR. ** represents $p \leq 0.01$. Only statistically significant results are shown.

Supplemental Figure 19

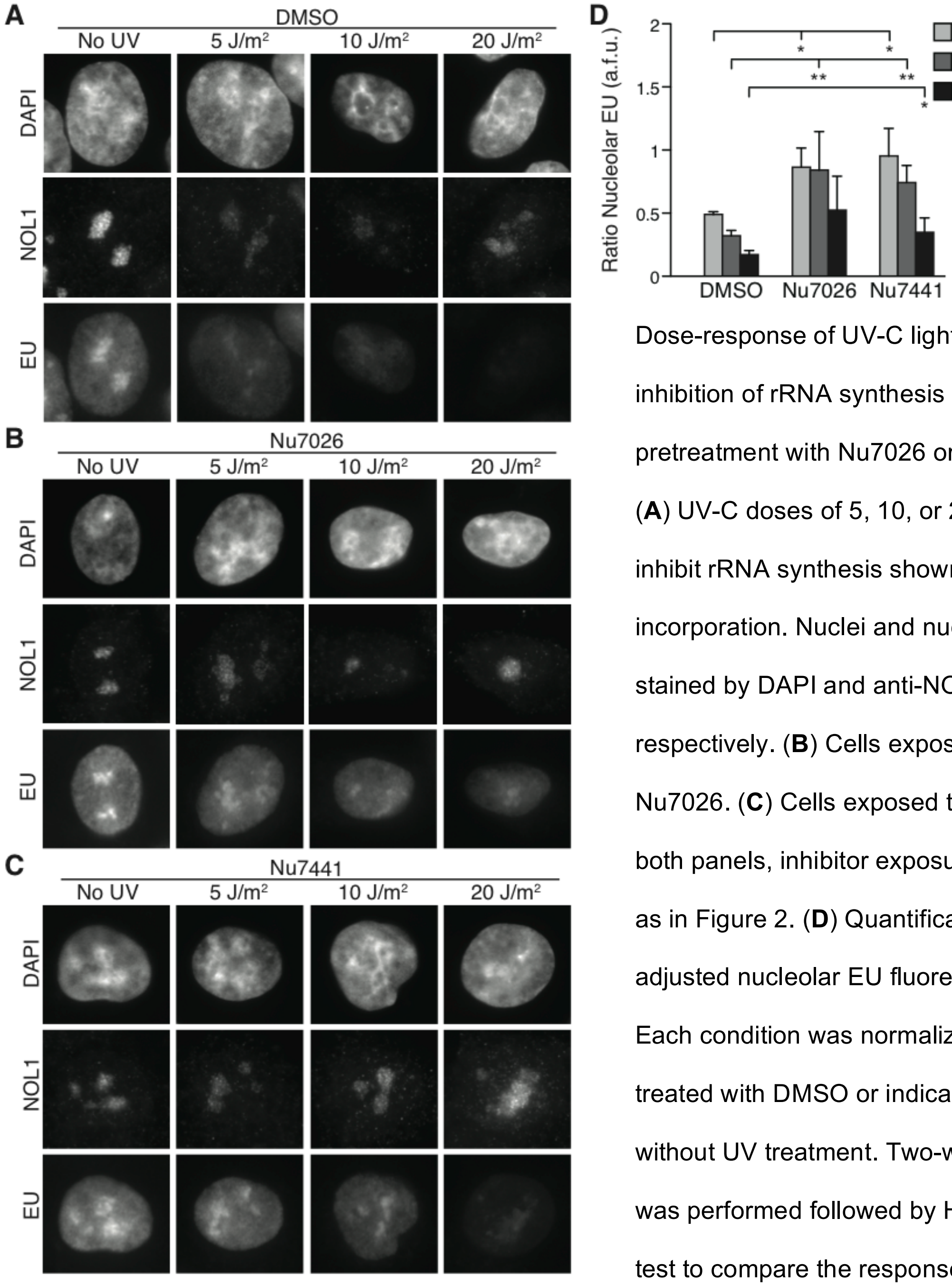

Dose-response of UV-C light-induced inhibition of rRNA synthesis following pretreatment with Nu7026 or Nu7441. (**A**) UV-C doses of 5, 10, or 20 J/m^2 inhibit rRNA synthesis shown by EU incorporation. Nuclei and nucleoli were stained by DAPI and anti-NOL1, respectively. (**B**) Cells exposed to Nu7026. (**C**) Cells exposed to Nu7441. In both panels, inhibitor exposure was done as in Figure 2. (**D**) Quantification of adjusted nucleolar EU fluorescence. Each condition was normalized to cells treated with DMSO or indicated inhibitors without UV treatment. Two-way ANOVA was performed followed by Holm-Sidak's test to compare the response of cells exposed to inhibitors versus cells exposed to DMSO. *, ** represent $p \leq 0.05$, 0.01, respectively. Only statistically significant results are shown.

Supplemental Figure 20

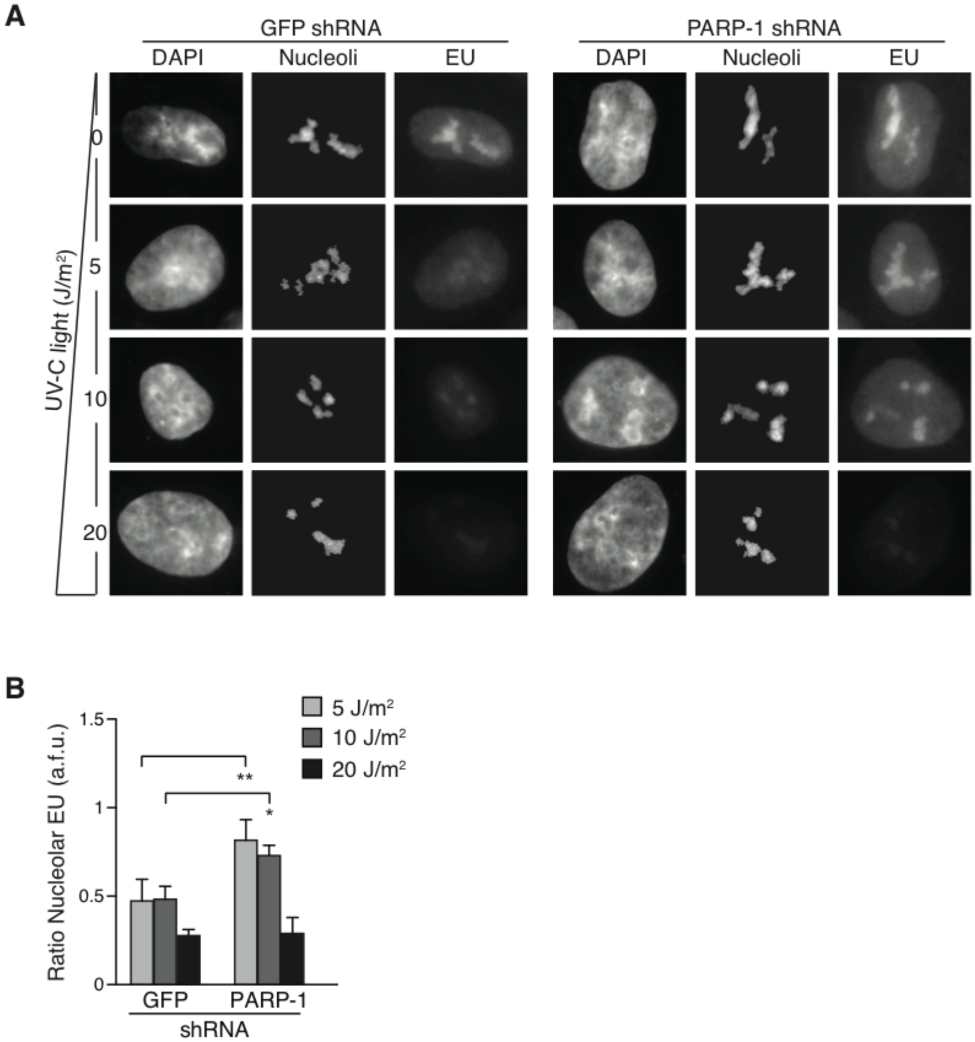

Dose-response of UV-C light-induced inhibition of rRNA synthesis in cells expressing GFP or PARP-1 shRNA. (**A**) UV-C doses of 5, 10, or 20 J/m^2 inhibit rRNA synthesis shown by EU incorporation. Nuclei were stained by DAPI, and CellProfiler was used to define Nucleoli from EU staining. Cells expressing shRNA to PARP-1 do not exhibit marked inhibition of rRNA synthesis at doses of 5 or 10 J/m^2. (**B**) Quantification of adjusted nucleolar EU fluorescence. Each condition was normalized to cells expressing the corresponding shRNA without UV treatment. Two-way ANOVA was performed followed by Holm-Sidak's test to compare the response of cells expressing GFP shRNA versus cells expressing PARP-1 shRNA at each dose of IR. * represents $p \leq 0.05$. Only statistically significant results are shown.

Supplemental Figure 21

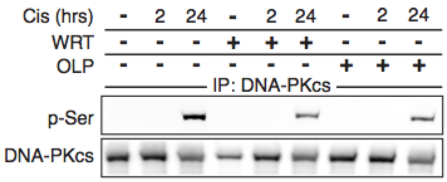

Immunoblotting analysis of samples after immunoprecipitation of DNA-PKcs following exposure to cisplatin (Cis), Nu7026 and Nu7441 as in Figure 2.

Supplemental Figure 22

A Original images input into CellProfiler

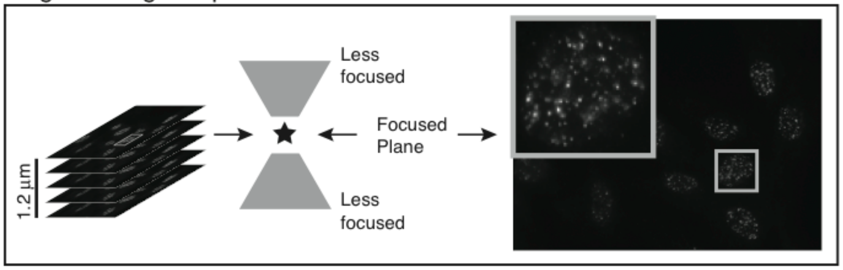

B Output: single projected image with distinct foci

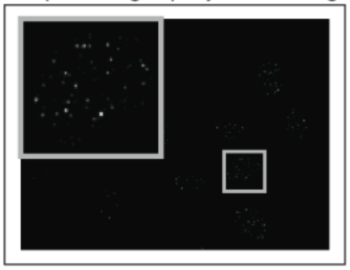

CellProfiler produces “projected” images for accurate quantification of foci. (**A**) During image acquisition, a stack of five images is taken. For each focused image, an additional two pictures were acquired above and below the most focused plane at an interval of 0.3 μm (z-stacking). The focused, middle image is shown with one nucleus blown up (gray box). (**B**) These five stacked images are input into CellProfiler, which generates a single “projected” image. This module eliminates pixels that vary the most from stack to stack; what appears in the top stack but not the bottom stack is rejected, while points that are consistent through the five stacks are retained. Much of the background and general fuzziness is removed from the image, making foci easier to quantify with CellProfiler’s shape-recognition modules.

Supplemental Figure 23

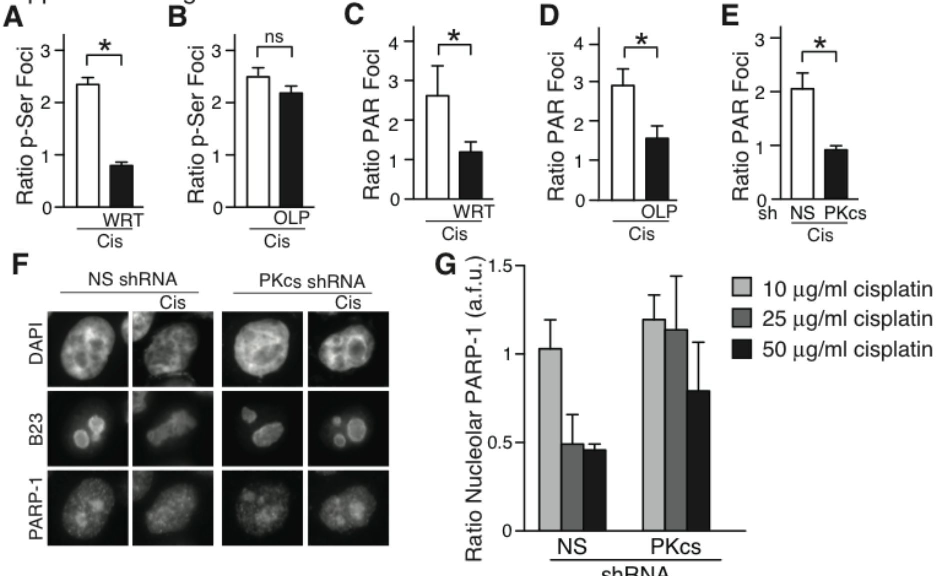

Regulation of PARP-1 by DNA-PK. Doses and exposure times of wortmannin and olaparib were identical to those used in Figure 2. (**A**, **B**) Quantification of the average number of DNA-PKcs p-Ser^{2056} foci (Ratio p-Ser foci) per nucleus in cells treated with 100 µg/ml cisplatin for 30 minutes following pretreatment with DMSO and (**A**) wortmannin (WRT) or (**B**) olaparib (OLP). (**C**, **D**, **E**) Quantification of the average number of PAR foci (Ratio PAR foci) per nucleus in cells treated with 100 µg/ml cisplatin for 30 minutes following pretreatment with DMSO or (**C**) wortmannin (WRT), (**D**) olaparib (OLP), or (**E**) non-silencing (NS) or DNA-PKcs (PKcs) shRNA. Each condition was normalized to cells exposed to DMSO or expressing the NS shRNA without cisplatin treatment. One-way ANOVA was followed by Holm-Sidak's test to compare responses to cisplatin of cells exposed to wortmannin or olaparib or expressing DNA-PKcs shRNA versus cells exposed to DMSO or expressing NS shRNA. * represents $p \leq 0.05$ for the tests indicated; ns indicates non-significant result ($p > 0.05$). (**F**) Nucleolar PARP-1 is lost from nucleoli after 2 hours of 25 µg/ml cisplatin treatment in cells expressing NS shRNA but not in cells expressing DNA-PKcs shRNA. Nuclei are shown stained with DAPI, and B23 is used as a marker of nucleoli. (**G**) Quantification of adjusted nucleolar PARP-1 fluorescence. Two-way ANOVA tested the response of cells expressing DNA-PKcs shRNA versus cells expressing NS shRNA. Statistical significance was found ($p \leq 0.05$) between the cell populations that expressed NS versus DNA-PKcs shRNA.

Supplemental Figure 24

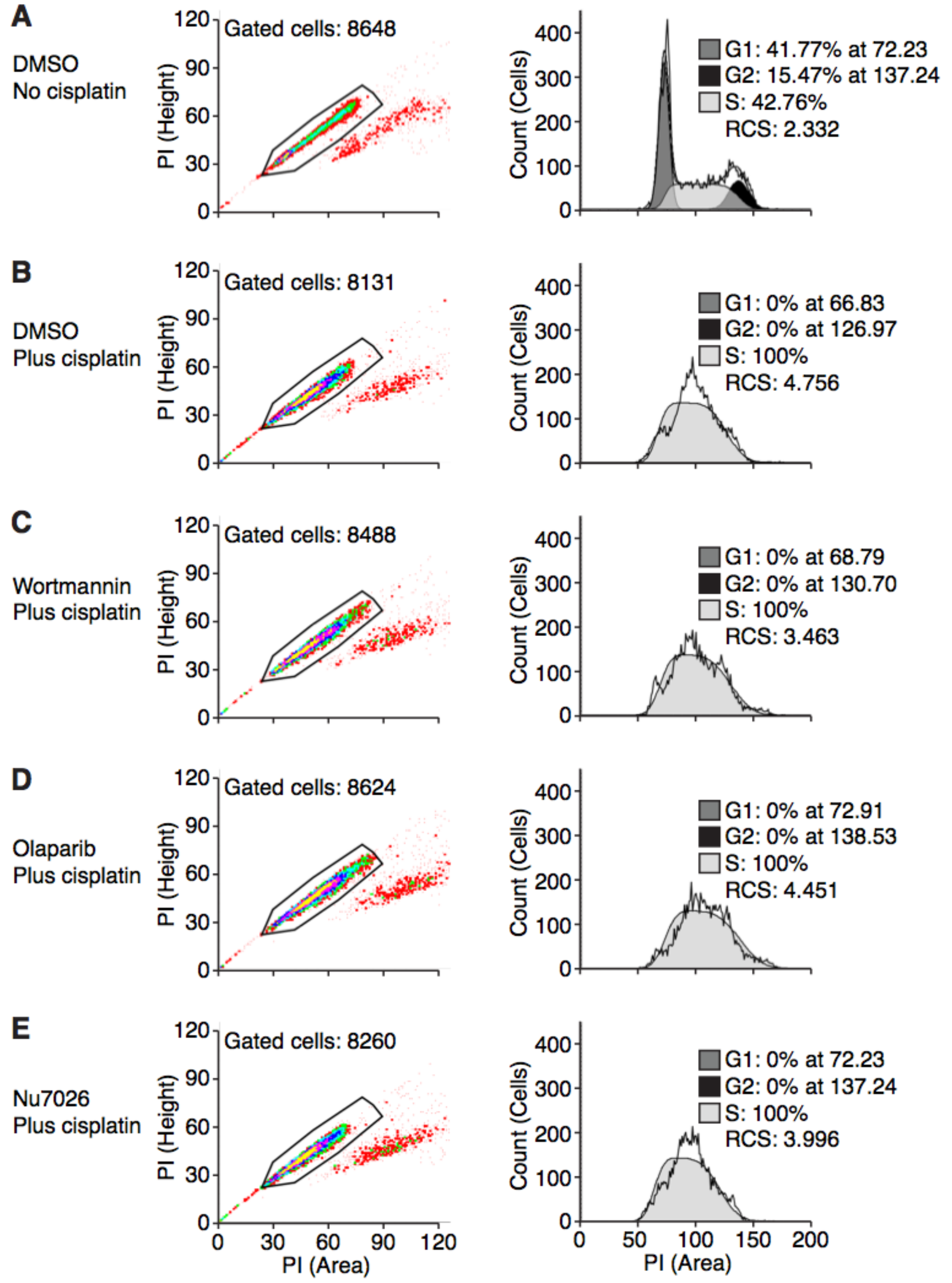

Flow cytometry analysis of (**A**) cells exposed to DMSO but not cisplatin. (**B**, **C**, **D**, **E**) Cells exposed to cisplatin following treatment with (**B**) DMSO, (**C**) wortmannin, (**D**) olaparib, and (**E**) Nu7026. Cell treatments were performed as in Figure 2. Gating of cells (10,000 total) is identical throughout samples. Percentages of cells in particular phases of the cell cycle were determined by peaks fitted to the control sample by ModFit LT. The best fit was applied to every condition. The reduced chi-square value (RCS) demonstrates quality of fit.
